# The neuromuscular junction is a focal point of mTORC1 signaling in sarcopenia

**DOI:** 10.1038/s41467-020-18140-1

**Published:** 2020-09-09

**Authors:** Daniel J. Ham, Anastasiya Börsch, Shuo Lin, Marco Thürkauf, Martin Weihrauch, Judith R. Reinhard, Julien Delezie, Fabienne Battilana, Xueyong Wang, Marco S. Kaiser, Maitea Guridi, Michael Sinnreich, Mark M. Rich, Nitish Mittal, Lionel A. Tintignac, Christoph Handschin, Mihaela Zavolan, Markus A. Rüegg

**Affiliations:** 1 Biozentrum, University of Basel, Basel, Switzerland; 2 Department of Neurology, Neuroscience, Cell Biology, and Physiology, Wright State University, Dayton, OH USA; 3 Department of Biomedicine, Pharmazentrum, University of Basel, Basel, Switzerland

**Keywords:** Ageing, Metabolism, Skeletal muscle

## Abstract

With human median lifespan extending into the 80s in many developed countries, the societal burden of age-related muscle loss (sarcopenia) is increasing. mTORC1 promotes skeletal muscle hypertrophy, but also drives organismal aging. Here, we address the question of whether mTORC1 activation or suppression is beneficial for skeletal muscle aging. We demonstrate that chronic mTORC1 inhibition with rapamycin is overwhelmingly, but not entirely, positive for aging mouse skeletal muscle, while genetic, muscle fiber-specific activation of mTORC1 is sufficient to induce molecular signatures of sarcopenia. Through integration of comprehensive physiological and extensive gene expression profiling in young and old mice, and following genetic activation or pharmacological inhibition of mTORC1, we establish the phenotypically-backed, mTORC1-focused, multi-muscle gene expression atlas, SarcoAtlas (https://sarcoatlas.scicore.unibas.ch/), as a user-friendly gene discovery tool. We uncover inter-muscle divergence in the primary drivers of sarcopenia and identify the neuromuscular junction as a focal point of mTORC1-driven muscle aging.

## Abstract (web summary)

mTORC1 expression is increased during ageing of muscle, and on the other hand, its activation promotes muscle hypertrophy. Here, the authors assess whether mTORC1 has positive or negative effects on ageing, and show that its long-term inhibition preserves muscle mass and function and neuromuscular junction integrity, whereas muscle-specific activation is associated with sarcopenia.

## Introduction

Rapid advances in treating life-threatening, age-related diseases, such as cancer and cardiovascular disease, have extended human lifespan in many countries, but exposed other age-related diseases, including sarcopenia, the age-related loss of muscle mass and strength. Sarcopenia constrains physical activity, degrades the quality of life, and is a major burden on society^1^.

Recently, nine processes involved in aging were proposed^2^, namely cellular senescence, stem cell exhaustion, genomic instability, telomere attrition, loss of proteostasis, deregulation of nutrient sensing, epigenetic alterations, mitochondrial dysfunction, and altered intracellular communication. Each biological process fulfils three hallmark criteria: (1) it occurs during normal aging, (2) intensifying the process accelerates aging, and (3) dampening the process delays aging. Overactivity of the mammalian (or mechanistic) target of rapamycin complex 1 (mTORC1) is central to many of these processes^3^, and dampening mTORC1 activity by its allosteric inhibitor rapamycin is one of the most effective interventions to prolong life^4^. However, mTORC1 activity is also required for muscle hypertrophy^5,6^. Therefore, there is concern that suppressing mTORC1 to extend lifespan could be at the expense of skeletal muscle function, thereby extending the “poor-quality” period of life^7^.

In previous work, we have shown that mTORC1 activity must be finely balanced in skeletal muscle. Constitutive, muscle-specific depletion of mTOR or the mTORC1 component raptor induces severe myopathy^8,9^. Likewise, specific depletion of raptor in muscle progenitors severely impacts muscle development and is prenatally lethal^10^. On the other hand, constitutive, skeletal muscle-specific knockout of tuberous sclerosis complex 1 (TSC1), an upstream inhibitor of mTORC1, in mice (TSCmKO) leads to sustained activation of mTORC1 and increased protein synthesis. However, rather than developing hypertrophic muscles, TSCmKO mice experience dysregulated proteostasis and develop a late-onset myopathy^11,12^. In rodents, mTORC1 activity is high in sarcopenic compared to adult muscle^13–17^, and the same has been seen in human biopsies^18^. Finally, rapamycin ameliorates muscle function in a number of muscular dystrophies^19,20^. Thus, it remains an open question as to whether activation or inhibition of mTORC1 could counteract sarcopenia.

Here, we demonstrate that long-term rapamycin treatment is overwhelmingly positive in aging skeletal muscle, preserving muscle size, function, and neuromuscular junction (NMJ) integrity. Interestingly, responsiveness to rapamycin differs between muscles, suggesting that the primary drivers of age-related muscle loss may differ between muscles. To dissect the key signaling nodes associated with mTORC1-driven sarcopenia, we create a comprehensive multimuscle gene expression atlas from (1) adult (10-months old), (2) geriatric (30-months old), and (3) geriatric, rapamycin-treated mice using mRNA-seq. We integrate these data with gene expression profiles of muscle from TSCmKO mice and synaptic and extra-synaptic regions. We identify the NMJ as a focal point of skeletal muscle responses to aging and mTORC1 signaling, and create SarcoAtlas, a comprehensive, publicly available (https://sarcoatlas.scicore.unibas.ch/) resource to further our understanding of the molecular mechanisms involved in sarcopenia. We conclude that overactivity of skeletal muscle fiber mTORC1 fulfils the three criteria necessary to be a hallmark of sarcopenia, and thereby firmly establish the TSCmKO mouse as a model of accelerated sarcopenia.

## Results

### Rapamycin attenuates sarcopenia

**Fig. 1:**
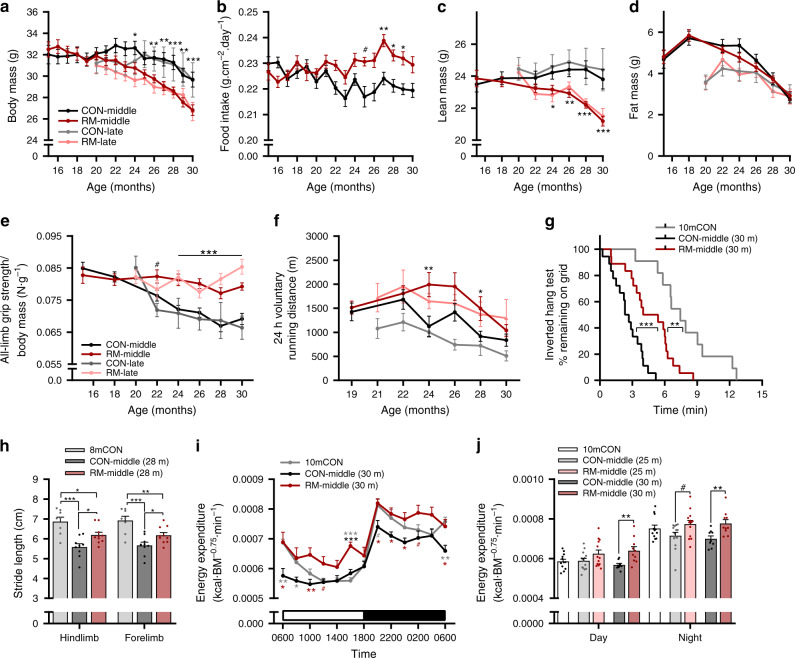
Rapamycin slows age-related decrements in whole-body muscle function and metabolism. **a** Body mass for mouse groups receiving rapamycin (~4 mg kg^−1^ day^−1^) or control diet beginning at 15 months (CON-middle and RM-middle) or 20 months (CON-late and RM-late) of age; *n* = 18 (CON-middle), 17 (RM-middle), 5 (CON-late), and 9 (RM-late) mice. **b** Mean daily food intake for middle-aged groups; *n* = 18 (CON-middle) and 17 (RM-middle) mice. Bimonthly recordings of whole-body lean (**c**) and fat mass (**d**); *n* = 18 (CON-middle), 17 (RM-middle), 6 (CON-late), and 9 (RM-late) mice. **e** All-limb grip strength normalized to body mass; *n* = 19 (CON-middle), 18 (RM-middle), 6 (CON-late), and 9 (RM-late) mice. **f** Twenty-four hours of voluntary running-wheel distance; *n* = 16 (CON-middle), 15 (RM-middle), 6 (CON-late), and 9 (RM-late) mice. Note that improvements by rapamycin were similar, irrespective of the time of treatment onset. **g** Kaplan–Meier plot for the inverted grip-hang test performed prior to endpoint measures at 30 months of age for the middle-aged group; *n* = 11 (10mCON), 18 (CON-middle), and 18 (RM-middle) mice. **h** Gait analysis of forelimb- and hindlimb-stride length at 28 months of age for middle-aged groups; *n* = 8 (8mCON), 9 (CON-middle), and 10 (RM-middle) mice. **i** Whole-body metabolic analysis of energy expenditure normalized to body surface area reported every 2 h across one full day (white)/night (black) cycle in the month prior to endpoint measures and **j** mean day and night values recorded at 25 and 30 months of age for middle-aged groups; *n* = 12 (10mCON), 14 (25mCON), 13 (25mRM), 9 (30mCON), and 10 (30mRM) mice. Data are presented as mean ± SEM. For **a**, **b**, **e**, and **f**, both control groups (CON-late and CON-middle) and both RM groups (RM-late and RM-middle) were combined for statistical comparisons. Two-way repeated-measure ANOVA with Sidak or Tukey post hoc tests (**a**–**f**, **i**–**j**), Mantel–Cox log rank (**g**), and one-way ANOVA with Fisher’s LSD post hoc tests (**h**) was used to compare the data. *, **, and *** denote a significant difference between groups of *P* < 0.05, *P* < 0.01, and *P* < 0.001, respectively. # denotes a trend where 0.05 < *P* < 0.10. Colored asterisks refer to the group of comparison.

To examine the impact of long-term rapamycin treatment, male C57BL/6 mice were fed encapsulated rapamycin incorporated into a standardized AIN-93M diet at 42 mg kg^−1^ of food, corresponding to a dose of ~4 mg kg^−1^ day^−1^, starting at 15 months (termed CON-middle and RM-middle, respectively) or 20 months (CON-late and RM-late) of age. This dose of rapamycin extends the lifespan maximally in male C57BL/6 mice^21^. Importantly, we carefully monitored body mass and food intake, and implemented a controlled feeding regime to curb the typical weight gain and obesity resulting from overeating in sedentary C57BL/6 mice^22^. Control mice maintained their pretrial body mass until around 26 months of age, at which point body mass progressively declined (Fig. 1a and Supplementary Fig. S1A). Rapamycin-treated mice, irrespective of the treatment onset, steadily lost body mass from ~20 months of age, and were significantly lighter than control mice from 26 months onward. Food intake, normalized to body surface area, significantly declined across the trial period in CON-middle mice, but this decline was completely prevented by rapamycin (Fig. 1b and Supplementary Fig. S1A). Bimonthly MRI recordings of body composition showed that the accentuated loss of body mass in rapamycin-treated mice was the result of lean mass loss, rather than fat mass loss, which markedly declined in both control and rapamycin groups (Fig. 1c, d and Supplementary Fig. S1A).

To ensure that our intervention period spanned a measurable loss of muscle function in control mice consistent with the development of sarcopenia, we performed repeated measures of whole-body muscle function throughout the experiment. In CON-middle and CON-late mice, all-limb grip strength decreased by ~20% by the age of 30 months, while grip strength remained stable in rapamycin-treated mice (Fig. 1e). Similarly, voluntary wheel-running distance decreased ~40% in control mice, while rapamycin significantly improved the running distance at the ages of 24 and 28 months (Fig. 1f). Rapamycin significantly improved inverted grid-hang time, which was sharply lower in 30-month-old CON-middle mice compared to a 10-month-old control group (10mCON, Fig. 1g). Rapamycin also significantly curtailed the age-related reduction in stride length (Fig. 1h), without affecting other gait parameters (Supplementary Fig. S1B–I). Consistent with reductions in food intake, 30-month-old mice displayed lower whole-body energy expenditure, especially in the early morning and early evening (Fig. 1i–j), coinciding with low-activity levels (Supplementary Fig. S1J, K). Respiratory exchange ratio (RER, Supplementary Fig. S1L, M) was also lower, indicating greater proportional fat utilization in 30- than 10-month-old mice during the low-activity, early morning period. In contrast, rapamycin-treated mice maintained a youthful energetic profile, with higher energy expenditure and daytime RER than control mice at 30 months, and an associated increase in VO_2_ ml kg^−1^ min^−1^ (Supplementary Fig. S1N, O). Importantly, the magnitude of the ubiquitous reductions in skeletal muscle function and whole-body metabolism in 30-month-old mice compared to 10- to 20-month-old mice is consistent with the clinical definition of sarcopenia^23^. Thus, this extensive, repeated-measures approach conclusively demonstrates a substantial age-related loss of muscle function across the treatment period, and that rapamycin effectively blunts these changes. Survival rates in our study were relatively high, with 59% of control and 68% of rapamycin-treated mice reaching 30 months of age. This compares favorably with the previously reported median lifespan for male C57BL/6 mice of ~29 months^24^, and could be related to our specific experimental conditions, such as single caging, the AIN-93M diet, and carefully controlled feeding in control mice to avoid overeating.

**Fig. 2:**
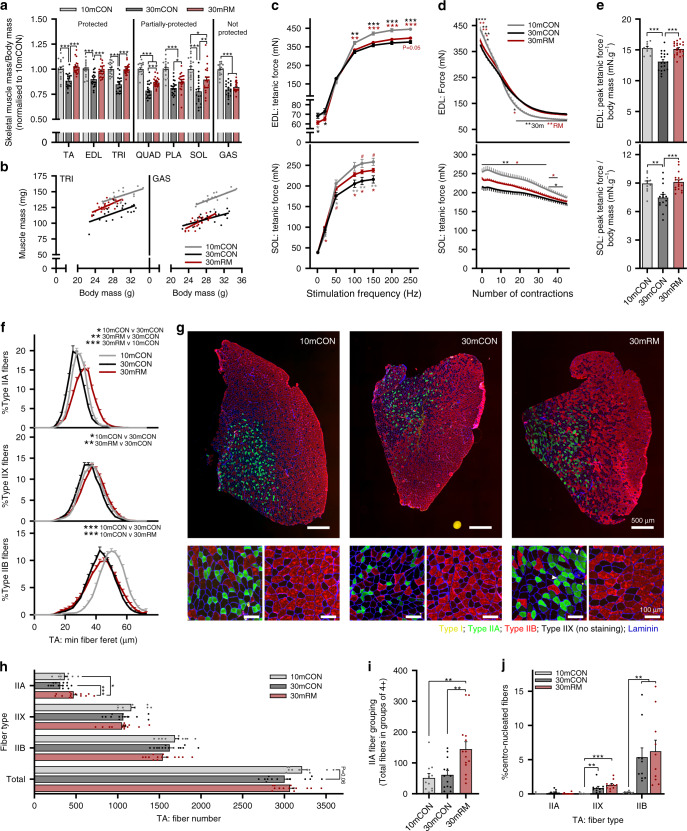
Rapamycin attenuates the age-related loss of muscle size and function. **a** Muscle mass for *quadriceps* (QUAD), *gastrocnemius* (GAS), *tibialis anterior* (TA), *plantaris* (PLA), *extensor digitorum longus* (EDL), *soleus* (SOL), and *triceps brachii* (TRI) was averaged across both limbs, normalized to body mass and then to 10-month-old control mice. **b** Scatterplot and linear regression of the relationship between body and muscle mass of a forelimb (TRI) and the non-rapamycin-responsive hindlimb (GAS) muscle. Isolated muscle function parameters, including (**c**) force-frequency curve, **d** fatigue response to multiple stimulations, and (**e**) peak force normalized to body mass for EDL (top panel) and SOL muscle (bottom panel). **f** Fiber-type-specific cross-sectional area analyzed on whole cross sections from TA muscle, stained with antibodies against type I (yellow), type IIA (green), and type IIB (red) fibers as well as laminin (blue), while fibers without staining were classified as IIX. **g** Representative images with magnification of IIA (green fiber, left bottom)-rich and IIB (red fiber, right bottom)-rich regions. White arrows indicate grouping (four or more neighboring fibers) of IIA fibers in rapamycin-treated mice. **h** Mean total and fiber-type-specific fiber number from whole TA cross sections. **i** Counts of IIA fibers in groups of four or more neighboring fibers, per cross section, and **j** the percentage of fibers with centralized nuclei. For **f**, between-group statistical comparisons were performed on the mean fiber minimum Feret. Group numbers for 10mCON are *n* = 17 (**a**, **b**), 10 (**c**, **e**: EDL), 11 (**c**, **e**: SOL), 8 (**d**: EDL), 9 (**d**: SOL), 11 (**f**–**i**), and 9 (**j**) mice, for 30mCON *n* = 20 (**a**, **b**), 19 (**c**–**e**: EDL), 15 (**c**–**e**: SOL), 13 (**f**–**i**), and 10 (**j**) mice, and for 30mRM *n* = 23 (**a**, **b**), 22 (**c**–**e**: EDL), 18 (**c**, **e**: SOL), 17 (**d**: SOL), 14 (**f**–**i**), and 10 (**j**) mice. Data are presented as mean ± SEM. One-way (**a** and **e**–**j**) or two-way repeated- measure (**c**, **d**) ANOVAs with Fisher’s LSD or Tukey’s (when ANOVA was not significant) post hoc tests were used to compare between data. *, **, and *** denote a significant difference between groups of *P* < 0.05, *P* < 0.01, and *P* < 0.001, respectively. Colored asterisks refer to the group of comparison.

As the endpoint results (i.e., at 30 months of age) for RM-middle and RM-late mice were very similar (Fig. 1a–f), we pooled the data for all further analyses (30mRM). Consistent with the age-related loss of whole-body function, mass of all measured muscles was significantly lower in 30-month-old mice (30mCON) compared to 10-month-old control mice (10mCON) when normalized to body mass (Fig. 2a) and for absolute mass (Supplementary Fig. S2A). Despite the ubiquitous age-related reductions in limb muscle mass, the effects of rapamycin were muscle-specific. Rapamycin protected against the age-related loss of relative muscle mass in the *tibialis anterior* (TA), *extensor digitorum longus* (EDL), and *triceps brachii* (TRI) muscles, partially protected relative mass in the *quadriceps* (QUAD), *plantaris* (PLA), and *soleus* (SOL) muscles, but did not protect relative mass in the *gastrocnemius* (GAS) muscle (Fig. 2a). The muscle-specific responses to rapamycin were also evident in absolute muscle mass, where triceps was significantly heavier and GAS significantly lighter in 30mRM than 30mCON mice (Supplementary Fig. S2A). Long-term rapamycin treatment also prevented the age-related increase in heart mass, and attenuated the increase in mass of nonfunctional tissues, such as the seminal vesicles, but induced severe testicular degeneration (Supplementary Fig. S2B), as previously observed^7^. Scatterplots showed a strong linear relationship between muscle and body mass in all groups. However, for the same body mass, muscle mass was lower in 30mCON compared to 10mCON mice (Fig. 2b and Supplementary Fig. S2C–F). In 30mRM mice, this relationship was shifted toward higher muscle mass in muscles protected by rapamycin (e.g., TA and TRI), but not in those that were not protected by rapamycin (e.g., GAS, Fig. 2b and Supplementary Fig. S2C–F).

As rapamycin-induced changes in non-skeletal muscle tissue (e.g., cardiac) and behavioral alterations could also affect muscle performance in mice, we next tested muscle function directly in the fast EDL muscle and slow SOL muscle. In line with the age-related loss of muscle mass and strength, tetanic force was significantly lower in 30mCON mice compared to 10mCON above a stimulation frequency of 100 Hz in EDL (Fig. 2c, upper) and 50 Hz in SOL (Fig. 2c, lower). Likewise, peak tetanic force was significantly lower in both the EDL (Supplementary Fig. S2G) and SOL (Supplementary Fig. S2M) in 30mCON compared to 10mCON. Rapamycin treatment significantly improved both EDL and SOL peak tetanic force, compared to 30mCON. The rapamycin-induced improvement in peak tetanic force was at least partially the result of improved muscle quality. Specific force, representing peak force normalized to muscle cross-sectional area, was unaltered by age, but significantly higher in 30mRM than both 10mCON and 30mCON in EDL and SOL (Supplementary Fig. S2H, N). In contrast, rapamycin reduced peak twitch force in EDL muscle without significantly altering other twitch properties (Supplementary Figs. S2I-K and S2O-Q). Fatigue resistance was higher in 30mCON and 30mRM than 10mCON mice in the EDL (Fig. 2d, upper and Supplementary Fig. S2L), but not SOL (Fig. 2d, lower and S2R). Importantly, in line with whole-body grip-strength measurements, when normalized to body mass, the age-related loss of EDL and SOL peak tetanic force was completely prevented by rapamycin (Fig. 2e).

Muscle fiber loss and type II fiber atrophy are prominent contributors to sarcopenia^25–27^. Indeed, we observed a strong reduction in minimum fiber Feret’s diameter of IIB and mild reductions in IIA and IIX fibers (Fig. 2f, g), and a trend for reduced total fiber number (Fig. 2h) in TA muscle from 30mCON compared to 10mCON mice. While IIB fiber atrophy was also a prominent feature in the TA muscle of 30mRM mice, a concurrent increase in size and number of IIA fibers was observed, such that minimum fiber Feret’s diameter and fiber number was significantly higher than in both 30mCON and 10mCON mice (Fig. 2f, h). Interestingly, higher IIA but not IIX or IIB fiber number strongly correlated with a larger fiber size in TA muscle from 30mCON and 30mRM (Supplementary Fig. S3A–C), indicating that IIB/X-to-IIA fiber conversion could explain IIA fiber enlargement in 30mRM. Analysis of a small subgroup of 21-month-old mice treated with control (21mCON) or rapamycin (21mRM) diets for 6 months showed that the enlargement of IIA and IIX fibers occurs in rapamycin-treated mice prior to measurable changes in muscle mass or fiber size (Supplementary Fig. S3D–H). IIA and IIX fiber size was significantly larger in TA muscle from 21mRM than both 21mCON and 10mCON mice (Supplementary Fig. S3F–H). Interestingly, a fast-to-slow fiber-type shift is also observed upon muscle-specific ablation of raptor in adult mice^28^, suggesting a direct effect of mTORC1 inhibition on the fiber-type composition of muscle. Similar, although less pronounced, fast-to-slow fiber-type shifts were also seen in 30mRM, but not 30mCON, compared to 10mCON in the EDL, TRI, and SOL (Supplementary Fig. S4A, C, E). Increased IIA fiber size was also observed in 30mRM compared to 30mCON in EDL and TRI, but not SOL (Supplementary Fig. S4B, D, F).

Fiber-type grouping is interpreted as compensatory reinnervation of denervated fibers by neighboring axons^29,30^. Motor neurons that innervate type IIA muscle fibers are more efficient at reinnervating denervated fibers than those innervating IIB fibers^26^. In 10mCON mice, IIA fibers were well dispersed within TA cross sections (Fig. 2g). While the number of “grouped” IIA fibers (defined as four or more neighboring IIA fibers) did not increase in 30mCON compared to 10mCON mice, rapamycin promoted a more than twofold increase in IIA fiber grouping (white arrows in Fig. 2g; quantification Fig. 2i). In addition, muscle degeneration/regeneration, as evidenced by the presence of centralized nuclei, strongly increased in the TA of 30mCON compared to 10mCON (Fig. 2j and Supplementary Fig. S5A). Interestingly, this age-related increase in centro-nucleated fibers was both fiber- and muscle-type specific. In the TA, the number of centro-nucleated fibers was significantly higher in both IIX and IIB fibers, but not IIA fibers, with the proportion of centro-nucleated IIB fibers approximately fivefold higher than IIX fibers. Age-related increases in centro-nucleated fibers were less pronounced in the EDL (Supplementary Fig. S5B), with trends observed in both IIA and IIB fibers, and nonexistent in the SOL (Supplementary Fig. S5C). Rapamycin did not alter the prevalence of centro-nucleated fibers in the TA, but higher numbers of centro-nucleated IIB fibers were observed in the EDL compared to 10mCON. Together, these results indicate that rapamycin improves muscle size by increasing the size and number of IIA muscle fibers, but does not reduce muscle degeneration/regeneration.

### Muscle fiber mTORC1 drives muscle wasting

**Fig. 3:**
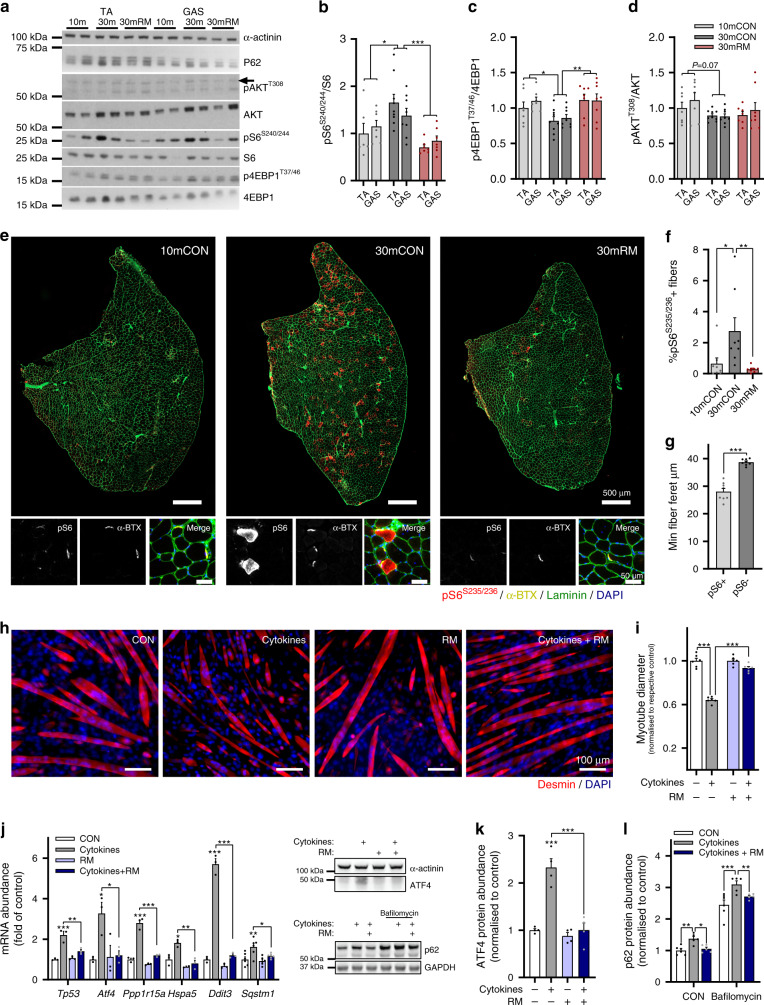
Rapamycin-sensitive mTORC1 activity increases in sarcopenic muscle fibers and drives muscle wasting in vitro. **a** Representative western blot analysis of AKT-mTORC1 pathway components in 10mCON, 30mCON, and 30mRM in both the *tibialis anterior* (TA) and *gastrocnemius* (GAS) muscle. Similar results were obtained for each protein across three separate gels with different samples. Quantification of western blots showing the abundance of phosphorylated protein normalized to total protein for (**b**) S6, (**c**) 4EBP1, and (**d**) AKT. **e** Representative images of TA cross sections stained for pS6^S235/236^ (red), α-bungarotoxin (neuromuscular junctions (NMJs), yellow), laminin (green), and DAPI (blue), and magnification of regions containing NMJs. Scale bars in full-section and enlarged images are 50 µm and 5 µm, respectively. **f** Quantification of the percentage of pS6^S235/236^-positive fibers in 10mCON, 30mCON, and 30mRM groups. **g** Quantification of minimum fiber Feret for pS6^S235/236^-positive and pS6^S235/236^-negative fibers in 30-month-old TA muscle. **h** Representative images of C2C12 myotubes incubated for 24 h in differentiation media (CON) or media containing 10 nM rapamycin, 20 ng ml^−1^ TNFα, and 100 ng ml^−1^ IFNγ (cytokines) or cytokines and rapamycin (cytokines + RM), and **i** quantification of myotube diameter. **j** RT-qPCR analysis of genes associated with ER stress and muscle wasting in C2C12 myotubes after 6 h of incubation in CON, cytokines, rapamycin, or cytokines+rapamycin. **k** Quantification and representative western blot image (left, upper) of ATF4 protein abundance after 6 h of incubation in differentiation media with or without cytokines and rapamycin (RM). **l** Quantification of p62 protein abundance and representative western blot image (left, lower) in differentiation media (CON) or media containing the autophagy-flux inhibitor bafilomycin (200 nM) after 4 h of incubation in media with or without cytokines and rapamycin (24 h). Group numbers for **b** are *n* = 6 mice for TA and seven for GAS (10mCON), eight for 30mCON, and seven for 30mRM. For **c** and **d**, *n* = 7 mice (10mCON), eight (30mCON), seven for TA, and eight for GAS (30RM). For **f**–**g**, *n* = 8. For **h**–**i**, *n* = 6 wells across two separate experiments. For **j**, *n* = 6 wells. For **k**, *n* = 4 wells. For **l**, *n* = 6 (CON) and five (cytokines and cytokines+RM) wells. Data are mean ± SEM. Two-way repeated-measure ANOVAs with Sidak post hoc tests (**b**–**d**, **i**), one-way ANOVAs with Fisher’s LSD (**f**), Tukey’s (**j**, **k**) post hoc tests, or student’s two-sided *t* test (**g**), were used to compare between data. *, **, and *** denote a significant difference between groups of *P* < 0.05, *P* < 0.01, and *P* < 0.001, respectively. Where 0.05 < *P* < 0.1, *P* values are reported.

It is still debated whether mTORC1 activity in skeletal muscle at high age is too high or too low^16,31^. However, recent evidence indicates that mTORC1 activity, as measured by the phosphorylation status of its downstream targets S6 kinase or S6, increases in old skeletal muscle^13–18^. So far, our data clearly demonstrate that systemic rapamycin administration attenuates the age-related loss of muscle mass and function. However, while mass in some muscles (e.g., TA) is preserved by rapamycin treatment, this is not the case in others (e.g., GAS). To test whether age- and rapamycin-mediated changes in mTORC1 signaling were also muscle specific, we next examined the phosphorylation of mTORC1 targets in both the TA and GAS using western blot analysis (Fig. 3a). We measured the phosphorylation status of S6 (S240/244) and 4EBP1 (T37/46), which are both involved in protein synthesis regulation, as well as PKB/AKT (T308), which is dampened by inhibitory feedback from the mTORC1 target S6K1 via IRS1^32^. We also examined the levels of the autophagy marker p62, which accumulates in muscle upon mTORC1 activation^11^. The ratio of phosphorylated to total S6 was significantly higher in 30mCON compared to 10mCON muscles, and was significantly suppressed by rapamycin treatment (Fig. 3b). Conversely, the ratio of phosphorylated to total 4EBP1 was significantly lower in 30mCON than 10mCON mice, while rapamycin restored the ratio (Fig. 3c). There was a tendency for lower pAKT/AKT (Fig. 3d) and higher p62 (Supplementary Fig. S6A) protein levels in 30mCON compared to 10mCON mice, but rapamycin did not significantly affect these parameters. However, no differences were observed between TA and GAS in any measured proteins, indicating that differences in mTORC1 activity cannot explain muscle-specific responses to rapamycin.

To determine whether increased mTORC1 activity was uniform in the muscle or localized to particular substructures, we stained TA cross sections with antibodies against pS6^S235/236^ and laminin (to outline muscle fibers), and used α-bungarotoxin to identify postsynaptic acetylcholine receptors (AChRs) at the NMJ. In previous work, we have shown that both pS6^S235/236^ and pS6^S240/244^ are equally sensitive to mTORC1 activation and suppression^19^. While pS6^S235/236^ staining was stronger in synaptic than extra-synaptic regions (Fig. 3e), as previously reported^33^, muscle fibers were largely negative for pS6^S235/236^ staining. However, in 30mCON mice, the number of pS6^S235/236^-positive muscle fibers increased four times compared to 10mCON mice (Fig. 3e, f). Rapamycin significantly reduced the number of pS6^S235/236^-positive fibers, without eliminating strong pS6^S235/236^ staining at the NMJ (Fig. 3e). In 30mCON mice, the mean minimum fiber Feret’s diameter of pS6^S235/236^-positive muscle fibers was significantly smaller than that of pS6^S235/236^-negative fibers (Fig. 3g), indicating atrophy in fibers with high mTORC1 activity.

To test the idea that mTORC1 signaling in muscle fibers contributes to muscle wasting, we investigated the effect of rapamycin on cultured C2C12 myotubes under basal conditions and in response to inflammatory cytokines. A chronic low-grade upregulation of circulating pro-inflammatory cytokines (e.g., TNFα, IL6, and CRP) is a common feature of sarcopenia^34^, and suppressing inflammation attenuates sarcopenia in rodents^35^. To establish the role of rapamycin-sensitive mTORC1 signaling under muscle-wasting conditions, we incubated C2C12 myotubes in media containing 20 nM of tumor necrosis factor-alpha (TNFα) and 100 nM interferon-gamma (IFNγ), collectively called “cytokines,” for 24 h with or without 10 nM of rapamycin. TNFα and IFNγ synergistically drive myotube wasting^36^. After 24 h, cytokine-treated myotubes were ~40% thinner than vehicle-treated cells, while 10 nM of rapamycin was sufficient to protect the myotubes from wasting (Fig. 3h, i). Myotubes in differentiation media treated with 10 nM rapamycin were thinner than control cells (Supplementary Fig. S6B), resulting from impaired differentiation (Supplementary Fig. S6C) and myotube hypertrophy (Supplementary Fig. S6D) between differentiation days 5 and 6. Indeed, differentiation is strongly impaired in C2C12 cells incubated in differentiation media containing rapamycin (>1 nM) for 5 days (Supplementary Fig. S6E). Importantly, despite impairing myotube hypertrophy across the treatment period, absolute myotube size was larger in cytokine- and rapamycin-treated cells than cytokine-treated control cells (Supplementary Fig. S6B). Furthermore, 10 nM rapamycin effectively prevented the cytokine-induced activation of well-known muscle-wasting pathways (Fig. 3j, k). Consequently, the increase in ATF4 protein by cytokines was efficiently blocked by rapamycin (Fig. 3k). Similarly, rapamycin prevented the accumulation of the autophagy marker p62 (Fig. 3l). The reduction in p62 protein by rapamycin was the result of reduced p62 production and not enhanced autophagic flux as shown using the autophagy inhibitor bafilomycin (Fig. 3l). These results show that mTORC1 activity increases in old, atrophic muscle fibers, and rapamycin blunts cytokine-induced muscle wasting by blocking transcription of several cytokine-induced stressors.

### Overactive muscle mTORC1 causes sarcopenia-like NMJ changes

**Fig. 4:**
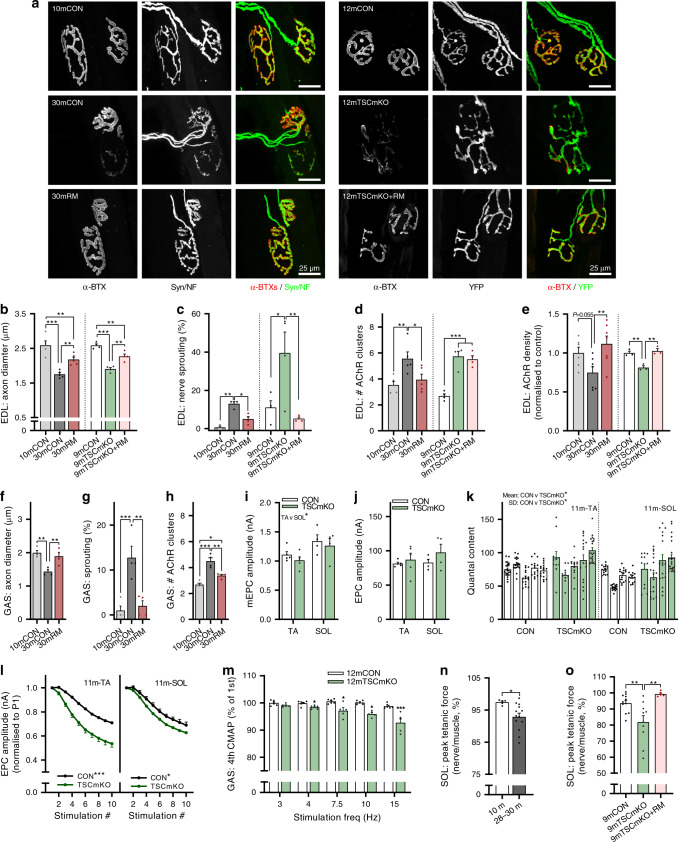
Overactive mTORC1 drives neuromuscular junction (NMJ) instability and impairs NMJ function. **a** Representative whole-mount images of the pre- (neurofilament/synaptophysin or YFP) and postsynapse (acetylcholine receptors (AChRs) stained using alpha bungarotoxin) in *extensor digitorum longus* (EDL) muscle for 10-month-old control (10mCON), 30-month-old (30mCON), and 30-month-old mice treated from 15 months with rapamycin (30mRM, left) and for 9-month-old control (9mCON), TSCmKO (9mTSCmKO), and TSCmKO + 4 weeks of rapamycin-treatment (TSCmKO+RM, right) mice. Morphological properties of whole-mount EDL NMJs, including **b** axon diameter, **c** the percentage of NMJs with sprouting axons, and **d** the number of postsynaptic AChR clusters for 9mTSCmKO and sarcopenic experiments. **e** Acetylcholine receptor density in EDL cross sections. **f**–**h** Morphological properties of whole-mount GAS NMJs for 10mCON, 30mCON, and 30mRM mice. In situ electrophysiological readouts of individual NMJ transmission properties in the *tibialis anterior* (TA) and *soleus* (SOL) muscle of 11-month-old TSCmKO mice and littermate controls, including (**i**) miniature end- plate current (mEPC), **j** end-plate current (EPC), **k** quantal content, and **l** EPC over repeated stimulations. **m** EMG recordings of compound muscle action potential (CMAP) rundown over four consecutive stimulations in the GAS of 9-month-old TSCmKO mice and littermate controls at stimulation frequencies between 3 and 15 Hz. Nerve- relative to muscle-stimulated peak tetanic force in SOL nerve-muscle preparations in (**n**) 10- (*n* = 5) and 28–30-month-old (*n* = 13) wild-type mice, and (**o**) 9-month-old control (*n* = 11), TSCmKO (*n* = 10), and TSCmKO mice treated with rapamycin for 4 weeks (*n* = 5). Group numbers for 10mCON are *n* = 5 (**b**, **d**), 3 (**c**), 6 (**e**), and 4 (**f**–**h**) mice. For 30mCON and 30mRM, *n* = 5 (**b**, **d**), 4 (**c**, **f**–**h**), and 7 (**e**) mice. For 9mCON, 9mTSCmKO, and 9mTSCmKO+RM, *n* = 4 (**b**–**d**) and 3 (**e**) mice. For 11mCON and 11mTSCmKO, *n* = 5 (TA: **i**–**l**) and 4 (SOL: **i**–**l**) mice. For **m**, *n* = 6 (12mCON) and 5 (12mTSCmKO) mice. For **n**, *n* = 5 (10m) and 13 (28–30 m). For **o**, *n* = 11 (9mCON), 10 (9mTSCmKO), and 5 (9mTSCmKO + RM) mice. Data are presented as mean ± SEM. One-way ANOVA with Fisher’s LSD post hoc test (**b**–**h**, **o**), two-way (**i**–**j**) or two-way repeated-measure ANOVA (**l**, **m**) with Fisher’s LSD post hoc test (**m**), or two-tailed independent student’s *t* test (**n**) were used to compare between data. *, **, and *** denote a significant difference between groups of *P* < 0.05, *P* < 0.01, and *P* < 0.001, respectively. Where 0.05 < *P* < 0.1, *P* values are reported.

NMJ instability is a common feature of aged muscle, and is thought to be a strong contributor to sarcopenia^37–40^. We have previously demonstrated that the sustained muscle fiber-specific mTORC1 activity in TSCmKO mice drives progressive muscle wasting and weakness reminiscent of sarcopenia^11,12^. More recently, we discovered that skeletal muscle fibers of TSCmKO mice (for *Tsc1* muscle knockout) show a strong impairment in the denervation-induced increase in AChR turnover, leading to rapid postsynaptic disintegration^33^. Together with the observations that mTORC1 signaling increases in 30mCON muscle, and dampening mTORC1 signaling with rapamycin ameliorates the sarcopenic features of aged mice, our previous denervation work led us to hypothesize that changes in mTORC1 signaling in postsynaptic muscle fibers may also affect NMJ stability. Typical morphological features of NMJ instability include axon thinning and sprouting, decreased postsynaptic AChR density, and fragmented postsynaptic structure^41,42^. To test this hypothesis, we performed a detailed evaluation of NMJ morphology using the publicly available NMJ-morph tool^43^ in 10mCON, 30mCON, and 30mRM mice, as well as 9-month-old control (9mCON) and 9-month-old TSCmKO (9mTSCmKO) mice with or without a 4-week treatment of rapamycin (2 mg kg^−1^ day^−1^, i.p). Age- and mTORC1-induced changes were examined using whole-mount images of EDL NMJs stained for the presynaptic nerve terminal and postsynaptic AChR clusters (Fig. 4a). Quantification revealed significant thinning of axons (Fig. 4b), an increased number of sprouting axons (Fig. 4c), and higher AChR cluster numbers, a measure of postsynaptic fragmentation (Fig. 4d). All these features were seen in 30mCON and 9mTSCmKO mice. In 30mRM mice, all age-related differences seen in 30mCON mice were ameliorated to levels similar to 10mCON mice (Fig. 4a–d). Similarly, a 4-week treatment of TSCmKO mice with rapamycin normalized axon diameter and sprouting, but did not affect the number of AChR clusters (Fig. 4a–d). In line with previous reports^37,44^, we observed lower density of postsynaptic AChRs in cross sections from EDL muscles of 30mCON (trend) as well as 9mTSCmKO mice, compared to adult control mice (Fig. 4e). In both cases, rapamycin treatment restored AChR density to control levels. Interestingly, although rapamycin did not protect the mass of GAS muscle from age-related loss, it did prevent age-related changes in NMJ morphology (Fig. 4f–h), indicating that rapamycin-induced improvements in NMJ structure are not responsible for the muscle-specific efficacy of the drug. To assess whether the structural changes at the NMJ would also affect neuromuscular transmission, we next used in situ electrophysiological recordings of single NMJs in fast-twitch TA and slow-twitch SOL muscle of 11-month-old TSCmKO (11mTSCmKO) and control (11mCON) mice. While the magnitude of miniature end-plate currents (mEPCs, Fig. 4i), resulting from spontaneous acetylcholine vesicle release, and evoked EPC (Fig. 4j) was not significantly altered, both the magnitude and variability (SD) of quantal content (EPC/mEPC, Fig. 4k) were significantly increased in the TA and SOL of 11mTSCmKO compared to 11mCON mice. Quantal content (i.e., the number of acetylcholine vesicles released in response to a single nerve action potential) is tightly regulated to ensure that each nerve action potential results in a muscle action potential, but must be balanced with the need for repeated stimuli and finite vesicle stores^41^. In line with findings in sarcopenic muscle, transmission fatigability in response to repeated stimulations increased in 11mTSCmKO mice (Fig. 4l). A deficit in neuromuscular transmission fatigue in electromyographic (EMG) recordings on the GAS of 12-month-old TSCmKO mice was also seen in compound muscle action potential (CMAP) measurements comparing the first to the fourth stimulation, starting at a stimulation frequency of 4 Hz (Fig. 4m). Transmission fatigue can result from decreased AChR density and therefore postsynaptic insensitivity. Finally, as an overall readout of NMJ efficiency, we used nerve-muscle preparations of the SOL muscle to compare the tetanic force generated by triggering action potentials in the nerve versus the muscle directly. In healthy, adult mice, nerve- and muscle-stimulated muscle force should be the same because of the safety factor of neuromuscular transmission^45^. Indeed, the ratio of force generated by nerve stimulation and by direct muscle stimulation was close to 1 in adult mice, while the ratio significantly dropped in both 28–30-month-old control mice (Fig. 4n) and in 9mTSCmKO mice (Fig. 4o). Four weeks of rapamycin treatment normalized the ratio in TSCmKO mice (Fig. 4o). These results strongly suggest that high mTORC1 signaling at high age is responsible for NMJ deterioration and hence to the development of sarcopenia.

### Muscle-dependent sarcopenic signaling responses to rapamycin

**Fig. 5:**
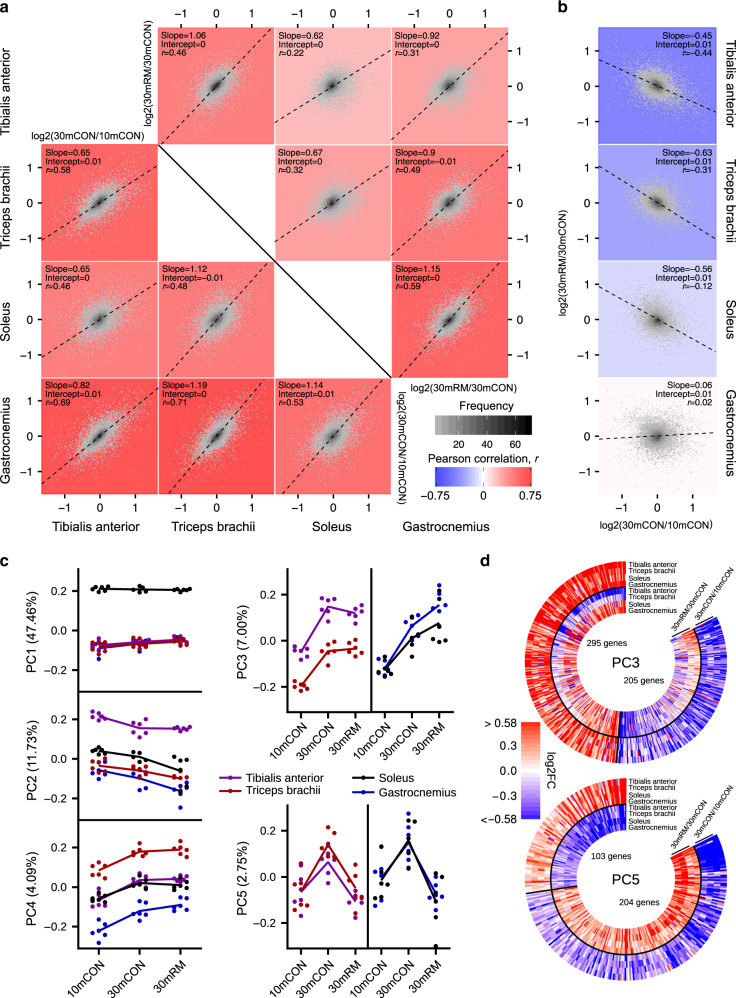
Rapamycin exerts pro- and anti-aging stimuli. **a** Pairwise comparisons of age- (30mCON/10mCON, under diagonal) and rapamycin-induced (30mRM/30mCON, above diagonal) gene expression changes between *tibialis anterior* (TA), *triceps brachii* (TRI), *soleus* (SOL), and *gastrocnemius* (GAS) muscles, and **b** pairwise comparisons of age- and rapamycin-induced gene expression changes within muscles with slope, intercept, and Pearson’s correlation coefficient (*r*) indicated. For **a** and **b**, each graph is a 2D-density plot with the plotting area divided into small fragments; the intensity of the gray color of each fragment represents the number of genes in that fragment. Black dashed lines correspond to directions of the highest variance (PC1) for comparisons with the slope “s” and intercept “i”. The color of the plotting area was defined by the strength of the Pearson correlation coefficient *r*. **c** Coordinates of principal components (PC1–5) for gene expression data collected in TA, TRI, SOL, and GAS in 10mCON, 30mCON, and 30mRM. *n* = 6 mice per group and muscle, except for SOL 30mCON where *n* = 5. Each dot corresponds to one muscle sample, from an individual animal. The numbers associated with the PCs indicate the fraction of the variance in transcript expression in samples along the corresponding PC. **d** Heatmaps of the changes in the expression of genes aligned with PCs 3 and 5, respectively. A gene was considered aligned with a PC if the absolute value of the Pearson correlation between the expression of the gene and PC coordinates was ≥0.4, and the absolute value of the *z* score of the projection of the gene expression on a PC was ≥1.96.

Diverse, muscle-specific gene expression profiles allow muscles to perform a wide range of different functions with different contraction properties^46^. We also observed a substantial difference in the responsiveness of muscles to aging, ranging from mild (EDL: −10.0 ± 1.8%, TA: −11.5 ± 1.5%, and TRI: −15.1 ± 1.8%; mean ± SEM) to severe (SOL: −22.2 ± 2.9%, QUAD: −21.4 ± 1.7%, and GAS: −20.4 ± 1.5%). Moreover, rapamycin was differentially effective in preventing loss of muscle mass (Fig. 2a). In an attempt to understand commonalities and differences between muscles in response to aging and rapamycin, we performed mRNA-seq on four muscles (GAS, TA, TRI, and SOL) from each of six mice for 10mCON, 30mCON, and 30mRM groups (i.e., a total of 72 muscles). In addition to their differential responses to aging and rapamycin, the four muscles were chosen to encompass fore- and hindlimb locations, slow and fast contraction properties, and anterior and posterior positioning. First, we investigated the age-related gene expression changes (i.e., log_2_(30mCON/10mCON)) between the four muscles (Fig. 5a, below diagonal) and observed moderate-to-strong correlations. Correlation strength was predominately related to fiber-type properties, with the strongest correlations observed between fast-twitch muscles (e.g., GAS vs. TRI, *r* = 0.71). Moderate correlations were still observed between the slow-twitch, SOL muscle and all three fast-twitch muscles (*r* = 0.46–0.53). Slopes, fitted to the direction of the highest variance (i.e., principle component 1), showed that the TA had the strongest age-related gene expression response (i.e., slope favors TA axis), followed by GAS, SOL, and TRI (Fig. 5a, below diagonal). In contrast to age-related gene expression changes, the correlation between muscles for rapamycin-induced gene expression changes (i.e., log_2_(30mRM/30mCON)) was unrelated to fiber type (Fig. 5a, above diagonal). The strongest correlation for rapamycin was between the physically co-localized, but fiber-type-distinct GAS and SOL muscle (*r* = 0.59), while correlations were the weakest between TA and both SOL (*r* = 0.22) and GAS (*r* = 0.31). Next, we compared age- (log_2_(30mCON/10mCON), *x* axis) and rapamycin- (log_2_(30mRM/30mCON), *y* axis) related gene expression changes for each muscle; negative correlations thus indicate the reversal of age-related changes by rapamycin (Fig. 5b). In line with the strong preservation of muscle mass at high age by rapamycin, age-related changes in mRNA expression were reversed by rapamycin in the TA and TRI. Likewise, in muscles partially protected (SOL) and not protected (GAS) by rapamycin, correlations were very weakly negative (*r* = −0.12, *s* = −0.56) and nonexistent (*r* = 0.02, *s* = 0.06, Fig. 5b), respectively. Hence, muscle-specific gene expression responses to rapamycin closely reflect rapamycin-induced changes in muscle mass (Fig. 2a).

To interrogate gene expression patterns in the different muscles in response to aging and rapamycin, we performed principal component (PC) analysis on data from all four muscles for 10mCON, 30mCON, and 30mRM mice (PC_10–30–30mRM_). PC1_10–30–30mRM_, PC2_10–30–30mRM_, and PC4_10–30–30mRM_ were associated with inherent differences between the muscles, independent of age and treatment (Fig. 5c and Supplementary Fig. S7A, B, D). In contrast, PC3_10–30–30mRM_ reflected age-related changes common to all four muscles, which were not affected by rapamycin in the TA and TRI, but exacerbated in the GAS and SOL (Fig. 5c, upper right). GO terms associated with PC3_10–30–30mRM_ were dominated by immune responses (e.g., neutrophil chemotaxis) and the NMJ (e.g., synaptic transmission, cholinergic; Supplementary Fig. S7C). PC5_10–30–30mRM_, on the other hand, reflected an “anti-aging” effect of rapamycin, consistent across all four muscles (Fig. 5c, lower right). GO terms associated with PC5_10–30–30mRM_ corresponded to extracellular matrix (ECM) components, which showed a remarkable age-related decrease (Supplementary Fig. S7E). We also visualized the log-fold changes in expression of genes aligned with PC3_10–30–30mRM_ and PC5_10–30–30mRM_ (Fig. 5d). This visualization reinforces the finding that age-related mRNA expression changes are consistent between muscles, while the effect of rapamycin is muscle specific. Together, these results indicate that the responsiveness of a muscle to rapamycin depends on the balance between pro- and anti-sarcopenic signaling.

### Signature of mTORC1-driven sarcopenic signaling

**Fig. 6:**
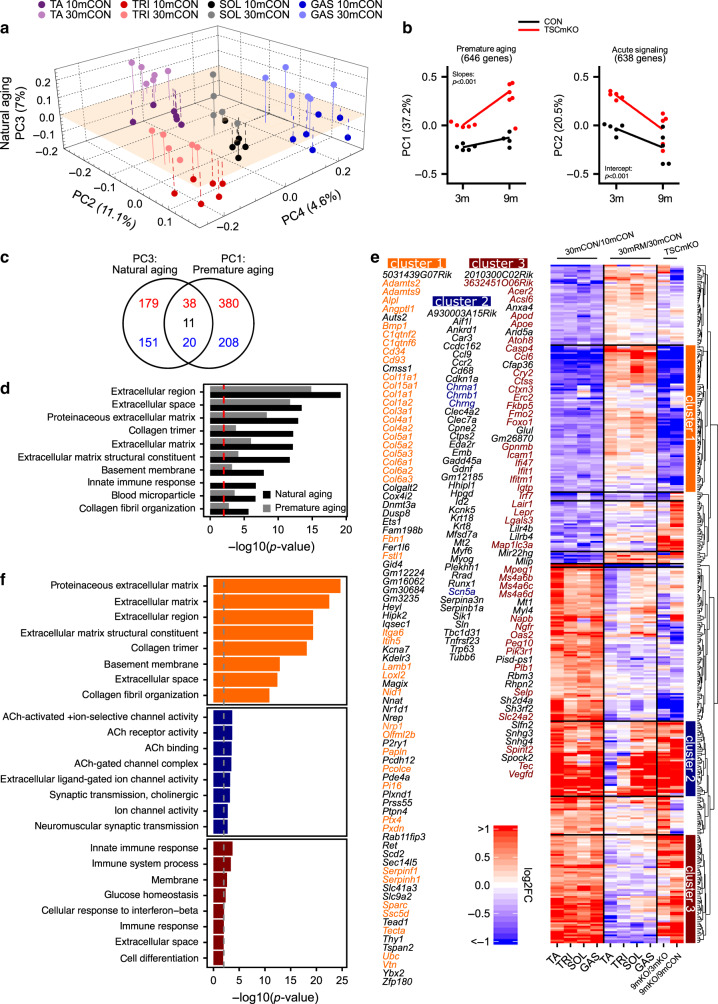
mTORC1 suppresses extracellular matrix (ECM) remodeling, promotes inflammation, and is involved in muscle responses to functional denervation. **a** Coordinates of principal components (PC2–4) for gene expression data (TPM) collected in *tibialis anterior* (TA), *triceps brachii* (TRI), *soleus* (SOL), and *gastrocnemius* (GAS) from 10mCON and 30mCON mice. **b** Coordinates of PC1 and PC2 for gene expression data (TPM) generated from *extensor digitorum longus* (EDL) muscle of 3- and 9-month-old TSCmKO and littermate control mice. The numbers associated with the PCs indicate the fraction of the variance in gene expression in samples along the corresponding PC. Each dot corresponds to one muscle sample, from an individual animal. Linear regression analysis was used to compare slopes and intercepts. **c** Overlap of genes aligned with PC3 from **a** (natural aging) and PC1 from **b** (premature aging). A gene was considered aligned with a PC if the absolute value of the Pearson correlation coefficient between the expression of the gene and PC coordinates was ≥0.4, and the absolute value of the *z* score of the projection of the gene expression on a PC was ≥1.96. Red and blue numbers represent increasing and decreasing genes, respectively, while black numbers represent genes oppositely regulated between TSCmKO and sarcopenia data sets. **d** Top-ten DAVID gene ontology terms enriched (*P* < 0.01) for genes aligned to natural aging PC3 and the enrichment of these terms for genes aligned with premature aging PC1. **e** Heatmap of changes occurring during aging in the expression of genes aligned with natural aging PC3, i.e., common age-related gene-expression changes (30mCON/10mCON) in all four muscles, along with fold changes in these genes for 30mRM/30mCON, 9mTSCmKO/3mTSCmKO, and 9mTSCmKO/9mCON. Hierarchical clustering based on the Euclidean distance of these changes rendered 10 gene clusters, including three prominent clusters, now referred to as clusters 1, 2, and 3. Genes associated with clusters 1, 2, and 3 are listed. **f** Top-eight DAVID gene ontology terms enriched (*P* < 0.01) for gene clusters 1, 2, and 3, respectively. Enrichment significance threshold was set at *P* < 0.01 (gray and red dashed lines). Genes from clusters 1 to 3 contributing to the top enriched terms are colored. For TSCmKO data set *n* = 5 mice per group. For the sarcopenia data set, *n* = 6 mice per muscle per group, except for SOL 30mCON where one data point was removed due to a technical error. A modified Fisher’s exact test was used to determine significance.

Next, we wanted to identify which age-related changes in gene expression could be ascribed to chronic activation of mTORC1 in muscle fibers. First, we identified a muscle-aging signature by performing PC analysis on all four muscles from the 10mCON and 30mCON groups (PC_10–30m_, Fig. 6a). PC1_10–30m_, PC2_10–30m_, and PC4_10–30m_ were once again associated with inherent differences between muscles, while PC3_10–30m_ represented age-related gene expression changes common to all muscles (natural aging PC3_10–30m_). Gene expression responses to rapamycin treatment in aged mice could result from mTORC1 inhibition in both muscle and non-muscle tissue. As the muscle phenotype of TSCmKO mice was highly similar to that of aged mice, but increased mTORC1 activity is restricted to skeletal muscle fibers, we next conducted mRNA-seq studies in EDL muscles from 3- (weak/no phenotype) and 9-month-old (severe phenotype) TSCmKO mice and their age-matched littermate controls. The first PC showed an aging-like pattern (premature aging PC1_TSCmKO_) with gene expression remaining stable in WT mice, but changing in TSCmKO mice between 3 and 9 months (Fig. 6b, left). PC2_TSCmKO_ represents early alterations that subside between 3 and 9 months in TSCmKO compared to control mice (Fig. 6b, right). Importantly, when comparing natural aging PC3_10–30m_ and premature aging PC1_TSCmKO_ commonly increasing (5.3-fold, *P* < 0.001) and decreasing (6.5-fold, *P* < 0.001) genes were over represented, and oppositely regulated genes (0.6-fold, *P* < 0.05, Fig. 6c) were under represented. Likewise, many of the top-ten enriched GO terms for natural aging PC3_10–30m_ were also enriched in premature aging PC1_TSCmKO_ (Fig. 6d). We next identified genes aligned with natural aging PC3_10–30m_ and expressed in all data sets (217 increasing and 171 decreasing), and performed hierarchical clustering of their log-fold changes in natural aging (30mCON/10mCON), rapamycin treatment (30mRM/30mCON), and premature aging (9mTSCmKO/3mTSCmKO and 9mTSCmKO/9mCON, Fig. 6e). Of the ten clusters with distinct expression patterns, three (now referred to as clusters 1–3) showed strong, consistent natural and premature aging-related gene expression changes and significantly mapped to GO terms (Fig. 6f). The first and third clusters contain genes whose expression decreased and increased, respectively, during natural and premature aging, and were reversed by prolonged rapamycin treatment. This suggests that muscle mTORC1 activity modulates gene expression changes in clusters 1 and 3. Consistent with our findings that rapamycin blocked cytokine-induced muscle wasting in C2C12 myotubes, genes associated with cluster 3 related to immune responses (Fig. 6f). Strikingly, genes in cluster 1 were highly enriched for structural components of the ECM, including all three major isoforms of collagen VI (*Col6a1–3*). Cluster 2 represents the muscle-specific “pro-aging” effects of rapamycin. Expression of the corresponding genes increased with both natural and premature aging, while rapamycin exacerbated these changes in muscles where mass was weakly, or not at all protected by rapamycin (i.e., GAS and SOL). Genes in this cluster mapped to components of the NMJ, and were remarkably enriched for known markers of functional denervation and NMJ instability^47–51^. Together, these data suggest that mTORC1 mediates age-related changes in ECM gene expression, immune response signaling, and denervation pathways. That systemic application of rapamycin exacerbates the latter pathway in specific muscles, indicates that the response depends on multiple inputs, some intrinsic and others extrinsic to the muscle.

### mTORC1-driven sarcopenic signaling converges on the NMJ

**Fig. 7:**
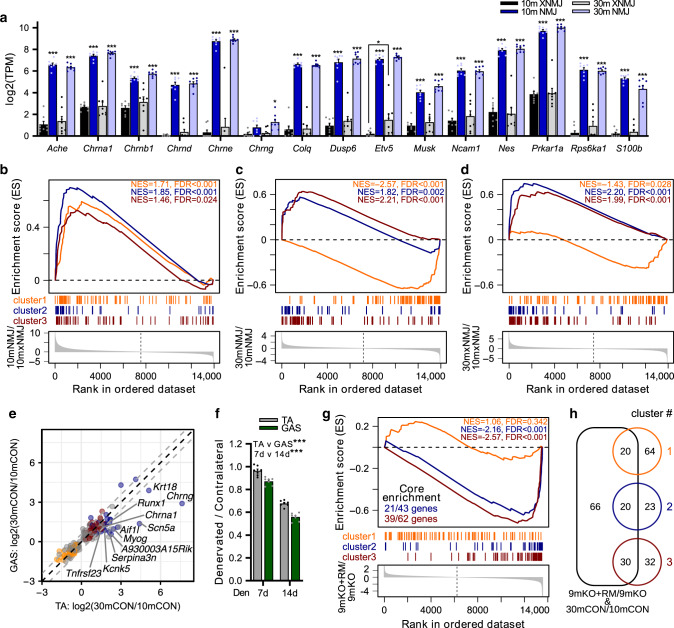
The neuromuscular junction (NMJ) is a focal point of mTORC1-driven sarcopenic signaling. **a** The expression of genes known to be enriched at the NMJ based on mRNA-seq data generated from synaptic (NMJ) and extra-synaptic (xNMJ) regions of TA muscle from 10-month-old (10m) and 30-month-old (30m) mice. NMJ and xNMJ samples were collected using laser-capture microdissection on α-bungarotoxin- stained cross sections. Gene set enrichment analysis (GSEA) comparing the expression of genes in (**b**) NMJ versus xNMJ regions of 10m mice. **c** NMJ regions of 30m versus 10m groups and **d** xNMJ regions of 30m versus 10m groups. The distribution of genes from clusters 1, 2, and 3 was examined in lists of genes ranked by the magnitude of changes between conditions. Significance was set at the false discovery rate (FDR) < 0.01. **e** Scatterplot of fold-change expression of genes aligned with the aging PC3 from Fig. 6a between 30m and 10m for *gastrocnemius* (GAS) and *tibialis anterior* (TA). Labeled genes show a > log_2_(1.5)-fold change between TA and all three other muscles. Genes from clusters 1, 2, and 3 are colored orange, red, and blue, respectively, while gray dots are genes aligned with aging PC3, but not belonging to clusters 1–3. **f** Muscle mass of TA and GAS muscles denervated for 7 or 14 days relative to the contralateral control leg. **g** GSEA comparing the distribution of genes from clusters 1–3 in the ranked list of gene expression changes between 9-month-old TSCmKO mice (9mKO) and 9-month-old TSCmKO mice subjected to 3 days of rapamycin (8 mg kg^−1^ day^−1^) treatment (9mKO+RM). For clusters significantly enriched, the number of genes reaching the “leading edge” threshold are indicated. **h** Venn diagram of genes differentially expressed between 9mKO+RM and 9mKO, and aligned with the aging PC3, and their overlap with genes in clusters 1–3. Group numbers are (**a**–**d**) 8, (**e**) 6, (**f**) 7, and (**g**) 5. For (**e**), each dot represents the log-fold change between means of mice in each group. Data are means ± SEM. Two-way ANOVAs (**a**, **f**) with Tukey’s post hoc test (**a**) were used to compare between data. *, **, and *** denote a significant difference between groups of *P* < 0.05, *P* < 0.01, and *P* < 0.001, respectively.

As rapamycin suppressed age-related morphological alterations to the NMJ, and TSCmKO mice display sarcopenia-like changes in NMJ transmission and morphology, our data pointed to the NMJ as a hotspot of mTORC1-driven muscle loss. Therefore, we next investigated whether the identified mTORC1-driven gene signatures (clusters 1–3 above) originate from cells residing at the NMJ. To do this, we identified NMJs in cross sections of TA muscle with α-bungarotoxin and isolated them by laser-capture microdissection. RNA-seq data from NMJ regions (NMJ) of adult and sarcopenic muscles were then compared with the results from non-NMJ regions (xNMJ). NMJ regions were highly enriched for genes known to be specifically expressed at the NMJ^52^ (Fig. 7a). Using a list of markers of muscle-resident cell types^53^, we found that glial (i.e., Schwann) cells were the only cell type consistently enriched in NMJ regions. Gene set enrichment analysis (GSEA) demonstrated that genes associated with clusters 1–3 were significantly enriched at the NMJ in adult (10m NMJ/10m xNMJ, Fig. 7b) mice. In NMJ regions, cluster 1 genes were significantly decreased, while cluster 2 and 3 genes were significantly increased in 30m compared to 10m muscle (Fig. 7c). Genes from clusters 2 and 3 also had increased expression in 30m xNMJ compared to 10m xNMJ regions, suggesting that age-related gene expression changes in these clusters were muscle-wide although expression was higher at the NMJ (Fig. 7d). On the other hand, the age-related depletion of genes associated with cluster 1 was more prominent in NMJ than non-NMJ regions, and may particularly compromise the stability of the NMJ and its ability to remodel following common age-related remodeling events, such as muscle fiber degeneration/regeneration and motor unit loss^42^.

Intermuscle differences in neural stability^39^ and compensatory reinnervation responses^38–40^ are thought to play a major role in determining the extent of muscle loss following age-related perturbations. We wondered whether the magnitude of age-related signaling (i.e., genes contributing to PC3_10–30m_) could explain between-muscle differences in the extent of age-related muscle loss (Supplementary Fig. S9A–L). While the extent of sarcopenia was lower in TA muscle than GAS or SOL (Fig. 2a), the expression of many genes involved in the muscle response to denervation (*Chrng*, *Myog*, *Runx1*, *Chrna1*, and *Scn5a*) was accentuated (>log_2_(1.5), Fig. 7e and Supplementary Fig. S9A–C). The marked upregulation of denervation-induced genes was the only signature differentiating the aging response in TA muscle from the other three muscles. Notably, all genes in this signature belonged to cluster 2, representing muscle-specific responses to rapamycin. Muscle-specific differences in gene expression and muscle phenotypes indicate that the enhanced age-related denervation response in TA is compensatory, while the enhanced rapamycin-related denervation response in GAS results from a more pronounced denervation stimulus. In line with this hypothesis, the TA lost significantly less muscle mass than the GAS 7 and 14 days after sciatic nerve resection, suggesting that it is better able to cope with denervation than GAS (Fig. 7f).

Finally, to determine whether gene expression changes in the mTORC1-driven muscle-aging clusters were a direct result of chronic skeletal muscle mTORC1 activity, and readily reversible, we treated 3- and 9-month-old TSCmKO mice and controls with rapamycin for 3 days, and performed mRNA sequencing on EDL muscles. GSEA showed a significant decrease in gene expression for half and two-thirds of clusters 2 and 3, respectively, but short-term rapamycin treatment could not restore the expression of genes in cluster 1 (Fig. 7g). Likewise, differential expression analysis showed that short-term rapamycin treatment reversed half of all genes in clusters 2 and 3, but only a quarter of genes in cluster 1 (Fig. 7h). This suggests that genes in clusters 2 and 3 are directly regulated by mTORC1, while genes in cluster 1 are not.

## Discussion

**Fig. 8:**
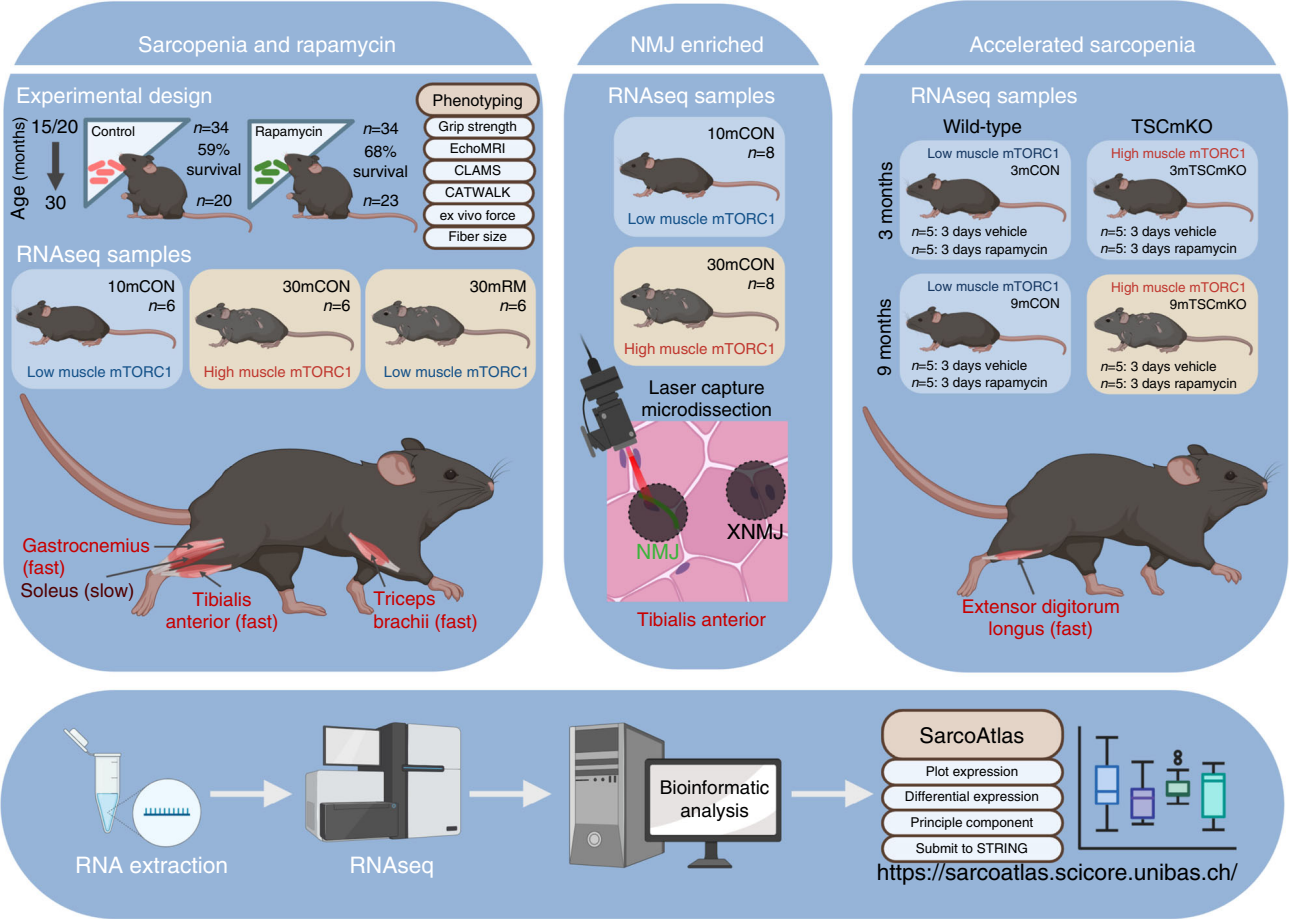
SarcoAtlas. A graphical representation of the experimental design, group numbers, treatment duration, and muscles analyzed for the three novel data sets generated, analyzed, and available through the user-friendly SarcoAtlas application (https://sarcoatlas.unibas.ch/). SarcoAtlas allows interested users to plot and extract expression data for a gene of interest, and perform principal component or differential expression analysis on any of the three novel data sets: (1) sarcopenia and rapamycin, (2) laser-capture microdissection of NMJ-enriched and non-NMJ (XNMJ) regions, and (3) TSCmKO model of accelerated sarcopenia. Furthermore, gene lists can be easily submitted to string to visualize interactions and investigate gene ontology. Figure created with BioRender.com.

Gene expression atlases (e.g., HAGR^54^) have greatly expanded our understanding of the regulatory mechanisms controlling organismal and tissue aging. However, muscle is chronically underrepresented in such atlases, and despite considerable between-muscle gene expression heterogeneity, generally restricted to a single muscle^46^. Here, we address this deficiency by establishing SarcoAtlas (https://sarcoatlas.scicore.unibas.ch/), a multi-muscle, multi-compartment, mTORC1-focused gene expression atlas based firmly on robust phenotypical characterization (Fig. 8).

While traditional wisdom stemming from short-term models of muscle hypertrophy and atrophy dictates that anti-sarcopenic interventions should focus on boosting mTORC1 activity^5,6^, evidence is mounting that efforts should rather focus on mTORC1 suppression. Here, we conclusively demonstrate that overactive skeletal muscle mTORC1 fulfils all criteria necessary to be considered a hallmark of sarcopenia^2^. First, mTORC1 activity is higher in atrophic myofibers of sarcopenic mice (Fig. 3). Second, sustained activation of mTORC1 in skeletal muscle fibers recapitulates age-related changes in NMJ stability (Fig. 4) and gene expression (Fig. 6). Third, long-term mTORC1 suppression by rapamycin significantly attenuates the age-related loss of skeletal muscle size and function (Figs. 1 and 2). Together, these data establish mTORC1 hyperactivity as a bona fide hallmark of sarcopenia and the TSCmKO mouse as a model of skeletal muscle fiber mTORC1-driven early sarcopenia.

Our data point to age-related NMJ instability as a focal point of mTORC1-driven sarcopenia. Maintenance of NMJ structure and transmission efficiency is crucial for preserving muscle function at high age^41^. Long-term rapamycin treatment preserved morphological indicators of NMJ stability (Fig. 4), while TSCmKO mice displayed NMJ instability and NMJ transmission changes comparable to old muscle, such as increased quantal content and transmission fatigue^55,56^. Age-related increases in quantal content are thought to compensate for postsynaptic insensitivity to preserve transmission efficiency^57^. However, increasing the number of ACh vesicles released in response to a single nerve action potential (i.e., the quantal content) can drive greater transmission depression during repeated stimulations^58^. Indeed, we observed increased EPC rundown (Fig. 4l, m) of individual NMJs from TA muscle, and increased CMAP depression in GAS muscle (Fig. 4n) from TSCmKO mice. Together with the observation that 4 weeks of rapamycin treatment restores NMJ stability in TSCmKO mice, these results strongly implicate sustained muscle fiber mTORC1 activity in age-related NMJ destabilization.

While the net effect of long-term rapamycin treatment on aging skeletal muscle was overwhelmingly positive, significant muscle-specific exacerbation of pro-aging signaling tempered its anti-aging effects. In line with our findings, 6 weeks of low-dose treatment with the rapalog RAD001 improved TA, but not GAS mass in old Sprague Dawley rats^15^. Tissue-specific effects of mTOR suppression on aging have also been noted in hypomorphic mTOR (mTOR^Δ/Δ^) mice, which expresses ~25% of wild-type mTOR protein^59^. Despite an overall positive phenotype, including improved muscle function and a 20% increase in lifespan, the mTOR^Δ/Δ^ mouse exhibits an exacerbated age-related loss of trabecular bone volume and increased superficial infections^59^. While chronic mTORC1 activation is clearly undesirable, acute, temporal increases in mTORC1 activity are important for many processes, including adaptive muscle growth^5^, as well as peripheral nerve regeneration and myelination^60,61^. As such, rapamycin may inhibit repair processes or remyelination in aging nerves, and thereby exacerbate some deleterious signaling in sarcopenic muscles.

The muscle-selective susceptibility to sarcopenia and responsiveness to rapamycin inspired us to ask whether age-related gene expression changes differ between muscles. While intermuscle diversity in gene expression was evident at both 10 and 30 months of age (PC2 and PC4, Fig. 6), age-related gene expression changes were remarkably conserved across four diverse limb muscles (PC3, Fig. 6 and below diagonal Fig. 5a), varying only in magnitude (Supplementary Fig. S9). The TA muscle displayed a markedly stronger “denervation” signaling response compared to the neighboring GAS or SOL muscle (Fig. 2a), including re-expression of AChRs, such as *Chrng*, coding for the embryonic AChRγ subunit (Fig. 7e). Expression of *Chrng* is strongly upregulated in non-synaptic regions of the muscle upon denervation^62^. Intriguingly, rapamycin heightened the denervation response in the GAS muscle (which is also not protected by rapamycin, Fig. 2a), but not in the rapamycin-protected TA muscle (Fig. 5). Importantly, the rapamycin-induced increase in AChR subunit (*Chrna1*, *Chrnb1*, and *Chrng*) gene expression in the GAS is accompanied by an increase in atrophic signaling, such as *Cdkn1a*, *Gadd45a*, and *Mt2*^49,63^, while the age-related enhancement of AChR expression in the TA is not, suggesting increased denervation, rather than an increased “compensatory” response to denervation. Indeed, the TA loses significantly less muscle mass than the GAS 7 and 14 days after sciatic nerve resection (Fig. 7f), and rapamycin-treated mice display pronounced fiber-type grouping, a sign of reinnervation, in the TA muscle (Fig. 2). Therefore, it is conceivable that the exaggerated denervation response (Fig. 7e) in the TA muscle allows it to better counteract age-related denervation and any exacerbation by rapamycin.

Our examination of gene expression patterns in sarcopenia points to three main processes: (1) diminished ECM remodeling, (2) increased functional denervation, and (3) increased inflammatory/immune signaling. Importantly, signaling in each of the three clusters followed a similar pattern in TSCmKO mice with accelerated, mTORC1-driven sarcopenia, indicating that muscle fiber mTORC1 activity contributes to these age-related changes.

Using laser-capture microdissection of synaptic and extra-synaptic regions coupled to RNA-seq, we observed a striking enrichment of mTORC1-driven, sarcopenia-signaling clusters in NMJ regions. Furthermore, the age-related depletion in the expression of ECM components in cluster 1 was more prominent in NMJ than extra-synaptic regions. While traditionally considered as a stable, structural support for skeletal muscle fibers, the expression of many components of the ECM responds to skeletal muscle remodeling. Resistance exercise promotes ECM component expression^64^, whereas experimental atrophy suppresses their expression^65^. Collagen gene expression also drops with age in *Caenorhabditis elegans*, and interventions that promote autophagy, such as calorie restriction (CR), rapamycin, and the ω-6 polyunsaturated fatty acids, γ-linolenic and arachidonic acid^66^, restore collagen expression and promote longevity^66,67^. The synaptic cleft of the NMJ comprises a thickened and specialized ECM, which promotes proper formation and maintenance of NMJ structure and transmission. Indeed, *Col6a1*-deficient mice display phenotypes consistent with NMJ destabilization^68^. Together, these findings indicate that mTORC1 influences sarcopenia through a complex network of muscle fiber-intrinsic and extrinsic signaling events hinging on the NMJ.

In conclusion, skeletal muscle fiber mTORC1 overactivity is a hallmark of sarcopenia, and the TSCmKO mouse represents a muscle fiber-driven model of accelerated sarcopenia. Our comprehensive gene expression atlas (https://sarcoatlas.scicore.unibas.ch/) of mTORC1-driven sarcopenia covers multiple muscles and multiple aging conditions, thus providing an invaluable resource to further our understanding of the molecular mechanisms driving sarcopenia. In skeletal muscle, contraction profiles, neural stability^39^, rates of cell turnover^69^, and intrinsic compensatory mechanisms^38–40^ can vary widely. Our multi-muscle data set highlights that the primary drivers of age-related muscle loss and therefore effective intervention strategies may differ between muscles.

## Methods

### Animal care

All procedures were performed in accordance with Swiss regulations for animal experimentation, and approved by the veterinary commission of the Canton Basel-Stadt. Male, C57BL/6JRj mice were purchased from the aging colony at Janvier Labs (Le Genest-Saint-Isle, France). TSCmKO transgenic mice and their genotyping were previously described^11,70^. Littermates, floxed for *Tsc1* but not expressing Cre recombinase, were used as controls.

### Rapamycin administration

Male C57BL/6JRj mice were kept on a 12-h light–dark cycle (6 am to 6 pm) at 22 °C (range 20–24 °C) and 55% (range 45–65%) relative humidity, and were acclimatized to individual housing and a control AIN-93M diet (TestDiet, 58M1-9GH6) containing 488 ppm Eudragit (Emtora) for 1 month before the start of experiments. In the week immediately prior to starting the experiment, body mass, food intake, grip strength, and body composition (via EchoMRI) were measured and used for balanced group selection. Each group (30mCON and 30mRM) contained mice with an almost identical mean and standard deviation for each measurement. 30mRM mice were switched to an AIN-93M diet containing 488-ppm encapsulated (Eudragit) rapamycin (TestDiet, 58M1-9GH7) for an active rapamycin composition of 42 mg kg^−1^. Food intake and body mass were measured weekly. For mice with a body mass greater than 34 g, we restricted daily food intake to the mean intake of the control group (3.1 g). Such restricted mice were given their food allotment daily at ~5 pm. This level of restriction prevented overeating, but did not induce behavioral alterations seen in calorie-restricted mice, such as anticipatory behavior, and mice were undistinguishable from their ad libitum- fed counterparts in all test parameters. Overeating was observed in around half of the control group, and a third of rapamycin-treated mice between 15 and 20 months. In rapamycin mice, food restriction was no longer necessary by 22 months of age, while the number of restricted control mice steadily dropped from 20 months of age, and was no longer required by 29 months of age. Mice given a restricted diet frequently did not eat their entire food allotment. The remaining food was weighed, and weekly food intake calculated.

### Body-composition analysis

Body composition, including fat and lean mass, was analyzed using an EchoMRI-100 (EchoMRI Medical Systems) in restrained, conscious mice.

### Whole-body muscle function

For voluntary running-wheel analysis, mice were first familiarized to wheel running for 3 days, and were then given free access to running wheels for a 24-h period every 2 months. Running activity was recorded every minute. Data are reported as the total distance run in the 24-h period. Mice consistently ran most in the early hours of the night and less during daytime hours. Activity patterns were not different between 10mCON, 30mCON, and 30mRM groups. For the inverted-hang test, mice were placed on a grid that was then slowly turned upside down and held in position ~40 cm over a box with a foam pad at the base, until the mouse could no longer hold onto the grid. Performance was taken as the best of three trials separated by at least 30 min. All-limb grip strength was measured by placing mice on a small grid attached to a force meter (Columbus Instruments). Once the mouse gripped firmly onto the grid with all four paws, the mouse was gently pulled horizontally at a consistent speed until the grasp was broken. Performance was measured as the median of three to five trials with at least 10 min of rest between tests. Trials where the mouse actively pulled on the grid while the test was underway, were discarded. The same researcher performed all grip-strength measurements at a similar time of day.

### Gait analysis

Gait analysis was performed using the Noldus CatWalk XT system^71^ following the manufacturer’s instructions (CatWalk XT 10.6 Reference Manual). Mice were trained to cross the illuminated walkway with at least three successful trials in the week prior to testing. Since testing was performed during daylight hours, mice were “warmed up” on a rotarod balance beam at a speed of 12 rpm for 10 min prior to completing the test. CatWalk XT software was used to analyze gait parameters. Stride length is the distance between successive paw placements. Swing speed and duration refer to the period where the paw is not in contact with the glass plate. The duty cycle refers to the stance phase as a percentage of the entire step cycle, i.e., stand + swing. Base of support refers to the distance between bilateral paws. Stand time refers to the period of time a paw is in contact with the glass between steps. A minimum of three compliant runs were completed for each mouse.

### Comprehensive laboratory animal monitoring system (CLAMS)

CLAMS (Columbus Instruments, Columbus, OH) was used to evaluate locomotor activity, energy expenditure, oxygen consumption (VO_2_), CO_2_ production (VCO_2_), and the respiratory exchange ratio (RER), which is the ratio of VCO_2_ to VO_2_. RER is dependent on energy substrate utilization, ranging from above 1 to 0.7, which indicates preferential use of carbohydrates and lipids, respectively. Locomotor activity was measured on *x*, *y*, and *z* axes using infrared beams. Energy expenditure was calculated using VO_2_ and RER values, and subsequently normalized to body surface area (body mass^−0.75^). Data were collected for 3 consecutive days, with the final 24-h period (6 am to 6 pm) used for analysis.

### In vitro muscle force

For in vitro muscle force measurements of the EDL and the SOL, muscles were carefully excised and mounted on the 1200 A Isolated Muscle System (Aurora Scientific, Aurora, ON, Canada) in an organ bath containing 60 mL of Ringer solution (137 mM NaCl, 24 mM NaHCO_3_, 11 mM glucose, 5 mM KCl, 2 mM CaCl_2_, 1 mM MgSO_4_, and 1 mM NaH_2_PO_4_) that was gassed with 95% O_2_, 5% CO_2_ at 30 °C. After defining the optimal length, muscles were stimulated with 15-V pulses. Muscle force was recorded in response to 500-ms pulses at 10–250 Hz. The fatigue of muscles was assessed by 6-min stimulation at 200 Hz for EDL and 120 Hz for SOL, respectively. To assess the nerve- versus muscle-stimulated force in vitro, SOL muscles were dissected with the sciatic nerve attached and mounted on the system described above. Muscles were stimulated either indirectly via the sciatic nerve through a suction electrode (with 4-V pulses), or directly via a pair of electrodes placed in the bath on both sides of the muscle^72^.

### Immunostaining of muscle cross sections

Muscles were mounted in optimal cutting temperature medium (O.C.T, Tissue-Tek) at resting length and snap-frozen in thawing isopentane for ~1 min before transfer to liquid nitrogen and storage at −80 °C. Muscle sections (10 µm) were cut from the mid belly at −20 °C on a cryostat (Leica, CM1950) and collected on SuperFrost Plus (VWR) adhesion slides and stored at −80 °C. Sections from each experimental condition were always mounted on the same slide to ensure accurate comparisons. For fiber typing, sections were blocked and permeabilized in PBS containing 10% goat serum and 0.4% triton X-100 for 30 min before being incubated for 2 h at RT in a primary antibody solution containing BA-D5, SC-71, BF-F3 and laminin (#11575, Abcam), and 10% goat serum. BF-F3, BA-D5, and SC-71 antibodies were developed by Prof. Stefano Schiaffino and obtained from the Developmental Studies Hybridoma Bank developed under the auspices of the National Institute of Child Health and Human Development, and maintained by the University of Iowa Department of Biology. Sections were washed four times for 10 min in PBS and then incubated in a secondary antibody solution containing DyLight 405 (#115-475-207, Jackson), Alexa568 (#A-21124, Invitrogen), Alexa488 (#A-21042, Invitrogen), Alexa647 (#711-605-152, Jackson), and 10% goat serum. Sections were then washed four times for 10 min in PBS and mounted with ProLong™ Gold antifade (Invitrogen). For fiber-type-specific centralized nuclei, type I fiber staining was replaced with DAPI staining in TA and EDL muscles, while type IIB fiber staining was replaced with DAPI, and DyLight 405 (#115-475-207, Jackson) was replaced with Alexa488 (#A21141, Invitrogen) secondary antibody in SOL muscle. For pS6 staining, sections were fixed in 4% PFA for 10 min prior to immunostaining. Primary antibodies used were pS6235/236 (#2211, Cell Signaling) and Laminin-2α (#11576, Abcam). Secondary antibodies were Alexa647 (#711-605-152, Jackson), Alexa488 (#112-545-003, Jackson), and α-bungarotoxin Alexa555-conjugate (#B35451, Invitrogen). Muscle sections were imaged at the Biozentrum Imaging Core Facility with an Axio Scan.Z1 Slide Scanner (Zeiss) equipped with appropriate band-pass filters. Fiji macros were developed in-house to allow an automated analysis of muscle fiber types or pS6-positive fibers (based on intensity thresholds) and muscle cross-sectional area (i.e., minimal Feret’s diameter, based on cell segmentation)^73^. All macros and scripts used in this study are available upon request.

### Protein extraction and western blot analysis

Cells were washed in ice-cold PBS and lysed in RIPA buffer (50 mM Tris-HCl, pH 8, 150 mM NaCl, 1% NP-40 (Roche), 0.5% sodium deoxycholate, 0.1% SDS, protease, and phosphatase inhibitors (Roche)). Dissected muscles were snap-frozen, pulverized in liquid nitrogen, and lysed in RIPA buffer with 20 mM EDTA, before sonication and incubation for 2 h at 4 °C. Insoluble material was removed by centrifugation (16,000 × *g*, 30 min, 4 °C). After adjustment of protein concentrations (determined by BCA assay) with RIPA buffer, samples were heated for 5 min at 95 °C in Laemmli buffer (0.1 M Tris-HCl, pH 6.8, 10% glycerol, 2% SDS, 0.04% bromophenolblue, and 1% β-mercaptoethanol). Proteins were separated on NuPAGE 4–12% Bis-Tris protein gels (Life Technologies) and transferred to nitrocellulose membranes. The membranes were then blocked in 5% BSA, 0.1% Tween20 in TBS for 45 min at RT, and then incubated overnight at 4 °C with primary antibodies, diluted in blocking solution. Membranes were then washed for 3 × 10 min in TBS before being incubated in secondary horseradish peroxidase-conjugated antibodies, diluted in blocking solution. After washing the membranes for 3 × 10 min in TBS, immunoreactivity was visualized using the KLP LumiGlo Chemiluminescence Substrate Kit (Seracare) with a Fusion Fx machine (Vilber). Protein abundance was quantified using FusionCapt Advance (Vilber) or ImageJ as mean gray value minus background and normalized to the housekeeping protein.

### Cell culture

Murine C2C12 myoblasts were cultured at 37 °C in an atmosphere of 5% CO_2_ in DMEM (61965026, Gibco) supplemented with 10% (v/v) fetal bovine serum (FBS, Biological Industries) and 1% (v/v) penicillin/streptomycin (Sigma, growth medium). After reaching confluence, low-passage cells (P6–P10) were incubated in DMEM supplemented with 2% (v/v) horse serum (Biological Industries) and 1% (v/v) penicillin/streptomycin (differentiation media), for 5–6 days to induce the formation of mature, multinucleated myotubes. To induce wasting, mature myotubes were incubated in differentiation media containing 20 ng ml^−1^ recombinant mouse tumor necrosis factor-alpha (TNFα, Gibco) and 100 ng ml^−1^ recombinant mouse interferon-gamma (IFNγ, Gibco) for 24 h. Rapamycin (LC Laboratories) was administered 1 h prior to the cytokine treatment. To investigate mTORC1 activity suppression by rapamycin, mature myotubes were incubated in 10 nM rapapmycin or 200 nM Torin1 (Millipore) for 24 h. Autophagic flux was measured by blocking the autophagy–lysosomal degradation system with 200 nM bafilomycin A1 (Med Chem Express) for 4 h before lysis.

### Myotube diameter

Myotubes were cultured in 12-well plates as described above. The medium was aspirated, and cells were washed twice for 5 min with Dulbecco’s PBS pH 7.4 (Sigma) to prevent detaching. Cells were then fixed with 3.7% PFA in D-PBS for 15 min and neutralized three times with MgCl_2_/CaCl_2_-free PBS (Gibco). Cells were blocked and permeabilized in PBS containing 3% (w/v) IgG-free BSA (Jackson) and 0.5% Triton X-100 (Sigma) for 1.5 h at RT. Afterward, myotubes were washed for 3 × 5 min with PBS. Primary antibody rabbit anti-desmin (1:300, ab15200, Abcam) was diluted in blocking solution (5% (w/v) IgG-free BSA in PBS) and incubated at 4 °C overnight. The next day, the primary antibody was removed, and cells were washed three times with PBS for 5 min. The secondary antibody Alexa488 (1:400, A-11034, Molecular probes) and DAPI (1:10,000) were diluted in blocking solution and incubated for 1.5 h at RT. Cells were then washed for 3 × 5 min with PBS and stored protected from light in PBS at 4 °C until imaging. Myotubes were analyzed, and images were taken with an inverted fluorescent microscope (Leica DMi 8) and the Leica Application Suite X (Leica) software. Four images were taken per well from predefined regions within each quadrant at a ×10 magnification. We defined myotubes as desmin^+^ cells containing at least three nuclei and displaying a tube-like structure. The diameter of 50–80 myotubes was measured in each well using the image analysis software Fiji, and the average diameter of each well was used for statistical analysis.

### RT-qPCR

For cell culture experiments, myotubes were lysed in RLT buffer (Qiagen), and RNA was extracted using the RNeasy^®^ Mini Kit (Qiagen), with Proteinase K and DNase treatment, according to the supplier’s instructions. RNA purity was determined using a Nanodrop ONEC (Thermo Scientific). cDNA was generated with the iScript^TM^ cDNA Synthesis Kit (Bio-Rad) using 500 ng of extracted RNA according to the supplier’s manual. cDNA samples were stored at −20 °C. RT-qPCR was performed in duplicate with the LightCycler 480 (Roche Diagnostics) instrument using LightCycler 384-well plates with sealing foil (Roche). The reaction volume of 10 μl contained FastStart Essential DNA Green Master Mix (2X, Roche), forward and reverse primers, and cDNA template (1:5 diluted). Primers were designed using Genious^®^10 software^74^, and specificity confirmed by the Basic Local Alignment Search Tool (BLAST)^75^. Potential hairpin formation, complementarity, and self-annealing sites were verified to be negative by OligoCalc^76^. The amplification of a single PCR product was confirmed with a melting-point dissociation curve, and raw quantification cycle (Cq) values were calculated by a LightCycler 480. Data were analyzed using the comparative Cq method (2^−ΔΔCq^). Raw Cq values of target genes were normalized to Cq values of a housekeeping gene (β-actin), which was stable between conditions, and then further normalized to the control group for ease of visualization. Primers used are as follows:

*Tp53*: forward—CACGTACTCTCCTCCCCTCAAT; reverse—AACTGCACAGGGCACGTCTT

*Atf4*: forward—AGCAAAACAAGACAGCAGCC; reverse—ACTCTCTTCTTCCCCCTTGC

*Ppp11r15a*: forward—GACCCCTCCAACTCTCCTTC; reverse—CTTCCTCAGCCTCAGCATTC

*Hspa5*: forward—TTCAGCCAATTATCAGCAAACTCT; reverse—TTTTCTGATGTATCCTCTTCACCAGT

*Ddit3*: forward—CCACCACACCTGAAAGCAGAA; reverse—AGGTGAAAGGCAGGGACTCA

*Sqstm1*: forward—AGGGAACACAGCAAGCTCAT; reverse—GACTCAGCTGTAGGGCAAGG

### Whole-mount NMJ staining and morphology quantification

For NMJ staining, muscle was fixed with 4% PFA for 1 h and cut into bundles. Muscle bundles were permeabilized with 2% Triton X-100, neutralized with 100 mM glycine, and blocked with blocking solution (3% BSA, 0.5% Triton X-100, and 0.01% NaN3 in PBS). Muscle bundles were then incubated with a mixture of primary antibodies against Synaptophysin and Neurofilament-200^73^ or 2H3/SV2^43^ in blocking solution overnight at 4 °C. After washing, the bundles were incubated in secondary antibody solution (Alexa555-conjugated α-bungarotoxin (B35451, Invitrogen) and DAPI). For the TSCmKO group, NMJ whole-mount analyses were performed in mice transgenically expressing YFP in motor neurons^77^. Therefore, staining of the presynapse was not performed in the TSCmKO mouse group. NMJ images of 512 × 512-frame size were acquired on a Zeiss LSM 700 confocal microscope with ×63 objective. Images were analyzed using ImageJ software following the workflow of “NMJ-morph,” as previously described^43^. The number of axonal inputs, NMJ terminal sprouting, and AChR fragments were counted on 3D reconstruction of confocal stacks with Imaris software.

### In situ electrophysiology

In situ electrophysiological recordings of single TA and SOL NMJs were done as previously described^78,79^. Briefly, 2–3-month-old mice were sacrificed using CO_2_ inhalation, and the TA and SOL muscles were removed and pinned in a sylgard-filled dish. After staining with 10 µM 4-(4-diethylaminostyryl)-N-methylpyridinium iodide (4-Di-2ASP, Invitrogen, Carlsbad, CA), NMJs were visualized using an epifluorescence microscope, and perfused at a speed of 3–6 ml per minute with an external solution containing (in mM) 118 NaCl, 0.7 Mg_2_SO_4_, 2 CaCl_2_, 3.5 KCl, 26.2 NaHCO_3_, 1.7 NaH_2_PO_4_, and 5.5 glucose (pH 7.3–7.4, 20–22 °C), equilibrated with 95% O_2_ and 5% CO_2_. End-plate currents were recorded using a two-electrode voltage clamp. The nerve to the TA or SOL muscle was stimulated via an extracellular Tungsten electrode (FHC Inc., USA). Muscle fibers were crushed away from the end-plate band to eliminate contractions upon nerve stimulation, and the holding potential was set at −45 mV.

### Repetitive nerve stimulation electromyography

The electromyographic properties of GAS muscle were measured using a Keypoint EMG machine (Meridian, Neurolite AG)^73^. Mice were anesthetized by intraperitoneal injection of ketamine (111 mg/kg, Pfizer) and xylazine (22 mg/kg, Streuli Pharma). The sciatic nerve was stimulated by trains of ten stimulations (0.04 ms of duration, 10 mA of amplitude, and 2-min pause between trains) at increasing frequencies from 3 Hz to 15 Hz. Action potentials were recorded in the GAS muscle by a needle electrode placed directly into the muscle belly (the reference electrode was inserted subcutaneously near the Achilles tendon). Decrement of CMAPs was calculated as percent between the first and fourth stimulation.

### Laser-capture microdissection and sequencing

The TA muscle was rapidly dissected and immediately immersed in 1 ml of Krebs–Ringer solution containing Alexa488-conjugated α-bungarotoxin (B13422, Molecular probes) for 1 h. Muscles were then snap-frozen in liquid nitrogen-cooled isopentane (Sigma-Aldrich) and partially embedded in 10% gum tragacanth (Sigma-Aldrich). Up to 30 serial 20-µm cross sections were cut at −20 °C on a cryostat (Leica) and mounted on RNase AWAY^®^-treated (Sigma-Aldrich) glass slides (Menzel). Sections were then dehydrated in 100% ethanol inside the cryostat chamber for 5 min and stored at −80 °C in 50-ml conical tubes containing several grams of desiccant (Silica Gel Orange, ROTH) to prevent tissue rehydration. Laser-capture microdissection (LCM) was performed using a PALM MicroBeam system (Zeiss). The microdissection process was visualized with an AxioCam ICc camera coupled to a computer and controlled by the PALM RoboSoftware (Zeiss). Synaptic regions were visualized with a ×20 objective, and ~300 synaptic regions (NMJ) and 300 extra-synaptic (XNMJ) regions equating to roughly 0.65 mm² were collected per sample. Selected areas were cut and catapulted by laser pulses, utilizing “autoLPC” mode, into opaque AdhesiveCap 500 PCR tubes (Zeiss). Tissue was pipette-homogenized and lysed for 5 min at RT with single-cell Lysis Buffer (635013, Clontech) supplemented with RNase Inhibitor (Roche Diagnostics) and then stored immediately at −80 °C until further processing. Importantly, the process was kept under 1 h to avoid RNA degradation by endogenous tissue RNAses. RNA library preparation was performed using the SMART-Seq v4 Ultra Low Input RNA Kit for Sequencing (Clontech).

### RNA extraction for aging-rapamycin and TSCmKO data sets

For the aging-rapamycin data set, snap-frozen TA, TRI, GAS, and SOL muscles from six mice per group were pulverized and lysed in RLT buffer (Qiagen) and treated with proteinase K (Qiagen). DNAse treatment and RNA extraction was performed with an automated iColumn 24 (AccuBioMed) using an AccuPure Tissue RNA Mini Kit (AccuBioMed). RNA purity and integrity was examined on a Bioanalyser (Agilent), while RNA concentration was determined using a Quant-iT™ RiboGreen™ RNA assay kit and Qubit flurometer (Invitrogen). Libraries were prepared with TruSeq Stranded mRNA HT Sample Prep Kit. Stranded, paired-end sequencing with 101 base-pair read length was performed on an Illumina HiSeq2500 platform. A single outlier in the 30mCON SOL group was identified and removed from further analysis based on a clear technical error. For the TSCmKO data set, poly(A) + RNA was extracted from whole EDL muscle lysates using the Dynabeads mRNA DIRECT Kit (61011, Invitrogen). Libraries were prepared according to the “Directional mRNA-seq sample preparation” protocol from Illumina. Libraries were subjected to stranded single-end sequencing with 50 base-pair read length on an Illumina HiSeq 2000 platform.

### Statistical analysis

All values are expressed as mean ± SEM, unless stated otherwise. Data were tested for normality and homogeneity of variance using a Shapiro–Wilk and Levene’s test, respectively. Data were analyzed in GraphPad Prism 8. Student’s *t* tests were used for pairwise comparisons, while one-way ANOVAs with Fisher’s LSD post hoc tests were used to compare between three groups, so long as the ANOVA reached statistical significance. Two-way ANOVAs with Sidak post hoc tests, or two-way repeated-measure ANOVAs for multiple recordings over time, were used to compare between groups with two independent variables. Both significant differences (*P* < 0.05) and trends (*P* < 0.1) are reported where appropriate.

### Differential expression analysis

Differential expression analysis was performed with EdgeR, available through the Bioconductor package^80^. Note that a gene was included in the analysis only if it had at least 1 count per million (CPM) in the number of samples corresponding to the minimum number of samples in each condition. Gene expression was considered statistically different between two conditions, if the false discovery rate (FDR) was less than 0.05^81^. Using EdgeR, log-fold changes in gene expression between pairs of all conditions were calculated and used for correlation analysis, heatmaps, and gene set enrichment analysis (GSEA).

### Gene set enrichment analysis

The distribution of gene sets in ranked gene lists was examined using GSEA^82^. Ranking was based on log-fold changes in the gene expression between two conditions of interest. Enrichment was considered significant if FDR was <0.01.

### Gene ontology analysis

To characterize the functions of genes identified by the analysis, we performed gene ontology (GO) analysis using Database for Annotation, Visualization and Integrated Discovery (DAVID)^83^. “GOTERM_BP_DIRECT,” “GOTERM_MF_DIRECT,” and “GOTERM_CC_DIRECT” categories were used for gene annotation, and GO terms with a *P* value < 0.01 were considered significantly enriched.

### Hierarchical clustering

Hierarchical clustering of genes was based on Euclidean distance between changes in the gene expression in notified conditions (see Fig. 6e).

### Shiny application

To make our high-throughput data sets and data analysis tools available for the research community, we developed an interactive web application using the R package Shiny (version 0.14.2, https://cran.r-project.org/web/packages/shiny/index.html). For each data set, the application supports gene expression plotting, differential expression analysis, principal component analysis, and aligning gene expression with principal components^80,84^. Moreover, the application can submit genes resulting from the analysis to STRING^85^ to further investigate protein–protein interactions and perform GO analysis. The application can be accessed through the following link: https://sarcoatlas.scicore.unibas.ch/

### RNA-seq data processing

RNA-seq reads were subjected to 3′ adapter and poly(A)/poly(T) tail trimming using Cutadapt v1.9.1^86^. The following 3′ adapter sequences were used to generate RNA-Seq libraries and then trimmed:

“Aging-RM” data set: Read1 5′-AGATCGGAAGAGCACACGTC-3′

Read2 5′-AGATCGGAAGAGCGTCGTGT-3′

“TSCmKO” data set: Read1 5′-TGGAATTCTCGGGTGCCAAG-3′

“NMJ” data set: Read1 5′-CTGTCTCTTATACACATCTCC-3′

Reads shorter than 20 and 30 nucleotides were discarded for single- and paired-end libraries, respectively. As the reference mouse transcriptome, we considered sequences of protein-coding transcripts with the support level 1–3 based on genome assembly GRCm38 (release 92) and transcript annotations from Ensembl database^87^. Kallisto v0.43.1 software was used for building the transcriptome index, with default options, and aligning filtered reads^88^. For aligning single-end RNA-Seq libraries, we used options “–single” and “-l” and “-s” corresponding to mean and standard deviation of fragment length, respectively, estimated for each sample by BioAnalyzer. For aligning strand-specific reads from “Aging-RM” and “TSCmKO” data sets, we used options “–rf-stranded” and “–fr-stranded,” respectively. For aligning filtered reads from all data sets, we used the option “–pseudobam,” which saves kallisto pseudoalignments to a BAM file.

Mapped reads were assigned to transcripts in a weighted manner: if a read was uniquely mapped to a transcript, then the transcript’s read count was incremented by 1; if a read was mapped to *n* different transcripts, each transcript’s read count was incremented by 1/*n*. Trimming 3′ adapter, indexing the reference transcriptome, mapping RNA-Seq libraries, and assigning mapped reads to transcripts was performed with Snakemake workflow framework^89^.

The expression of each transcript *t*_*i*_ was then estimated in transcripts per million (TPM) units by dividing its read count *c*_*i*_ by the transcript length *l*_*i*_ and normalizing to the library size:

1 $$t_i = \frac{{\frac{{c_i}}{{l_i}}}}{{\mathop {\sum }\nolimits_{j = 1}^{\# \,of\,tanscripts} \frac{{c_j}}{{l_j}}}} \cdot 10^6.$$

The expression of a gene was obtained by summing up the normalized expression of the transcripts associated with it. For every gene, read counts of transcripts associated with this gene were also summed up and further used for the differential expression analysis.

### Aligning gene expression with principal components

Gene expression data define a high-dimensional space, where each gene can be viewed as a point, whose coordinates are the expression levels in individual samples. Global relationships between samples can be uncovered by determining the directions in the sample space that capture most of the variation in the gene expression^84,90^. Here, we describe how we used this approach with the help of a toy data set consisting of three “samples” and 26 “genes” measured for each sample. Gene expression in these samples was randomly sampled from the multivariate normal distribution, such that 20 genes do not differ significantly in expression level between samples (*P* value of the *t* test ≥ 0.01), three have significantly lower, and three significantly higher expression in sample 3 compared to the other samples (*P* value of the *t* test <0.01). We visualized these data in Supplementary Fig. S10A, in the 3D space of samples, where each point corresponds to a gene. To identify samples with significantly different patterns of gene expression, as well as the genes that contribute most to this difference, we used singular-value decomposition (SVD).

First, we constructed a matrix with *m* columns (samples) and *n* rows (genes), such that each cell in the matrix contains the expression level of a particular gene in a particular sample in log scale (logTPM). Note that a gene was considered for the analysis only if it had at least 1 TPM in the number of samples corresponding to the minimum number of samples in each condition. To make the data comparable across samples and genes, we centered the columns and rows of the matrix by subtracting column and row means from each cell. Thus, the sum of the values within a column or row is zero, as depicted in Supplementary Fig. S10B. The resulting matrix was designated matrix *G* and subjected to SVD

2 $$G_{[n \times m]} = U_{[n \times m]} \cdot D_{\left[ {m \times m} \right]} \cdot V_{\left[ {m \times m} \right]}^T =[\vec u_1\,\vec u_2\, \cdots \vec u_m] \cdot diag\{ d_1,d_2, \ldots ,d_m\} \cdot [\vec v_1\,\vec v_2\, \cdots \vec v_m]^T,$$

where *D* is a diagonal matrix containing singular values *d*_1_, *d*_2_,…, *d*_*m,*_ *U* is a matrix of left singular vectors $\{ \vec u_i\} _{i = \overline {1,m} } \in {\Bbb R}^n$, and *V* is a matrix of right singular vectors $\{ \vec v_j\} _{j = \overline {1,m} } \in {\Bbb R}^m$ of the matrix *G*. The vectors $\{ \vec v_j\} _{j = \overline {1,m} }$ define the directions that capture most variance in the data set, in descending order, under the constraint that they are orthogonal. This property of vectors $\{ \vec v_j\} _{j = \overline {1,m} }$ can be explained by the association between SVD and principal component transformation, which is based on the factorization of the covariance matrix. Indeed, since the matrix *G* is mean centered, the $m \times m$ covariance matrix *C* of the gene expression data can be expressed as^91^

3 $$C = \frac{{G^TG}}{{n - 1}} = \frac{{V\,D\,U^T \cdot U\,D\,V^T}}{{n - 1}} = V\frac{{D^2}}{{n - 1}}V^T.$$

Thus, Eq. (3) provides the mapping between SVD and principal component analysis, as matrix *V* is the matrix of eigenvectors of the covariance matrix *C*, which are principal components (PCs) of the gene expression data in the space of samples (Supplementary Fig. S10C). The decomposition of the covariance matrix *C* also demonstrates that the proportion of variance in the data explained by PC $\vec v_j$ can be calculated by

4 $$\lambda _j = \frac{{d_j^2}}{{\mathop {\sum }\nolimits_{i = 1}^m d_i^2}}.$$

One is generally most interested in the PCs that explain the largest proportion of the variance. In our toy example, PC $\vec v_1$ explains about 85% of the variance in the data set (Supplementary Fig. S10D). The directions of the PCs are defined by their coordinates in the sample space—the more similar the coordinates, the more similar the samples. In our example, the coordinates corresponding to samples 1 and 2 of the PC $\vec v_1$ are similar to each other and differ strongly from the coordinate corresponding to sample 3.

We used a geometric approach to identify genes associated with a PC $\vec v_j$. Every gene *k* can be represented in the sample space by the vector $\vec g_k$ drawn from the origin to the point with coordinates corresponding to the *k*th row of matrix *G*. For representing the PC vector $\vec v_j$ we used its coordinates in the sample space derived above, and then calculated both the magnitude of the projections and correlations of the gene vectors with PC $\vec v_j$. Gene vectors aligned with $\vec v_j$ and with high absolute projection values on the PC $\vec v_j$ contribute most to the variance explained by PC $\vec v_j$. Supplementary Fig. S10E illustrates these concepts, showing projections of a representative gene vector (blue arrow) on PCs $\vec v_1$ and $\vec v_2$ as well as the angles between the gene vector and PCs.

Projection and correlation measures can be quantified using matrices *U*, *V*, and *D* from SVD of matrix *G* (see Eq. (1)). Projections of a gene vector $\vec g_k$ on PC vectors $\{ \vec v_j\} _{j = \overline {1,m} }$ correspond to a *k*th row of the *n* × *m* matrix *P*:

5 $$P = G\,V = U\,D\,V^T\,V = U\,D.$$

In this study, we considered a projection significant if its absolute *z*-score value was _≥_ 1.96. For our toy example, projections of gene vectors on PCs $\vec v_1$ and $\vec v_2$ are depicted in Supplementary Fig. S10F, G, respectively.

Pearson correlation between a gene vector $\vec g_k$ and a PC $\vec v_j$ was calculated as a cosine of the angle between these vectors:

6 $${\mathrm{cos}} \, \theta = \frac{{\vec g_k \cdot \vec v_j}}{{\left\| {\vec g_k} \right\| \cdot \left\| {\vec v_j} \right\|}} = \frac{{P_{kj}}}{{\left\| {\vec g_k} \right\|}} = \frac{{P_{kj}}}{{\sqrt {\mathop {\sum }\nolimits_{l = 1}^m P_{kl}^2} }}.$$

We considered a correlation between gene vector and PC as significant if the absolute value was ≥0.4. For the toy example, correlations between PCs $\vec v_1$ and $\vec v_2$ and gene vectors are depicted in Supplementary Fig. S10H, I, respectively. The figures indicate that significantly different genes in sample 3 have both significant absolute projections and correlations with the vector $\vec v_1$. These genes contributed most to the variance in our toy example.

### Reporting summary

Further information on research design is available in the Nature Research Reporting Summary linked to this article.

## Supplementary information

Supplementary Information

Reporting Summary

## Data Availability

Raw and processed RNA-seq data are available at Gene Expression Omnibus (GEO)^92^ (https://www.ncbi.nlm.nih.gov/geo/) under accession number GSE139214 (SubSeries GSE139204, GSE139209, and GSE139213). These data are also accessible using the web-based application, SarcoAtlas (https://sarcoatlas.scicore.unibas.ch/). Source data are provided with this paper.

## Source data

Source Data

## Code availability

Code is available upon request.
