# Supplementary material for: The neuromuscular junction is a focal point of mTORC1 signaling in sarcopenia
Source: Nat Commun. 2020 Sep 9;11:4510. doi: 10.1038/s41467-020-18140-1 (PMC7481251; doi:10.1038/s41467-020-18140-1)
Supplement: Supplementary file 1 — Supplementary Information [file 41467_2020_18140_MOESM1_ESM.docx]

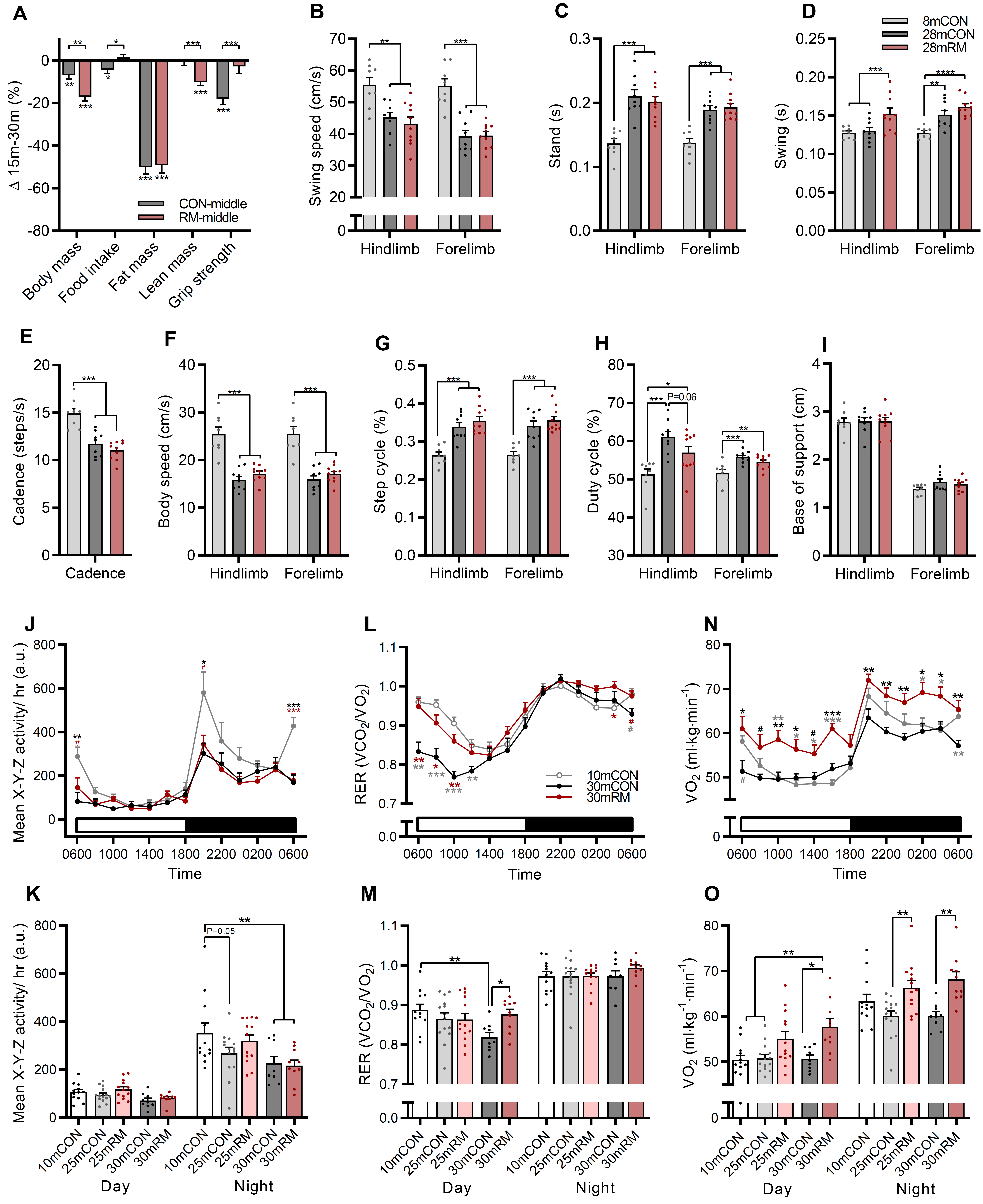


**Figure S1. Delta body composition and grip strength, gait analysis and whole-body metabolism.** (A) Delta changes from 15 to 30 months of age in body mass and composition, food intake and grip strength for control (CON-middle) and rapamycin-treated (RM-middle) mice. (B-I) Gait parameters of 8-month-old control mice (8mCON), 28-month-old control mice (28mCON) and 28-month-old mice treated with rapamycin starting at 15 month of age (28mRM). A detailed explanation of each parameter can be found in the extended methods section. Whole-body metabolic analysis of (J, K) mean locomotory activity, (L, M) respiratory exchange ratio (RER) and (N, O) oxygen consumption (VO_2_ml·kg^-1^·min^-1^) using the Comprehensive Laboratory Measuring System (CLAMS). Values are (J, L, N) 2 hour means across one full day (white)/night (black) cycle in the month prior to endpoint measures and (K, M, O) mean day and night values recorded at 25 and 30 months. Group numbers are (A) n=18 (CON middle) and 17 (RM middle), except for grip strength where n=19 (CON middle) and 20 (RM middle); (B-I) n=6 (10mCON), 9 (30mCON) and 10 (30mRM) and; (J-O) n=12 (10mCON), 14 (25mCON), 9 (30mCON), 13 (25mRM) and 10 (30mRM) mice. Data are presented as mean ± SEM. Two-tail independent (between groups) and paired (between time points) t-tests (A); One-way ANOVA with Fisher’s LSD post hoc test for fore and hind limbs (B-I) and; two-way repeated measures ANOVA with Tukey’s post hoc test (J-O) were used to compare between data. *, ** and *** denote a significant difference between groups of P<0.05, P<0.01 and P<0.001, respectively. Where 0.05<P<0.1, P values are reported or the trend is denoted by #. Color of the asterisks refers to the group of comparison.


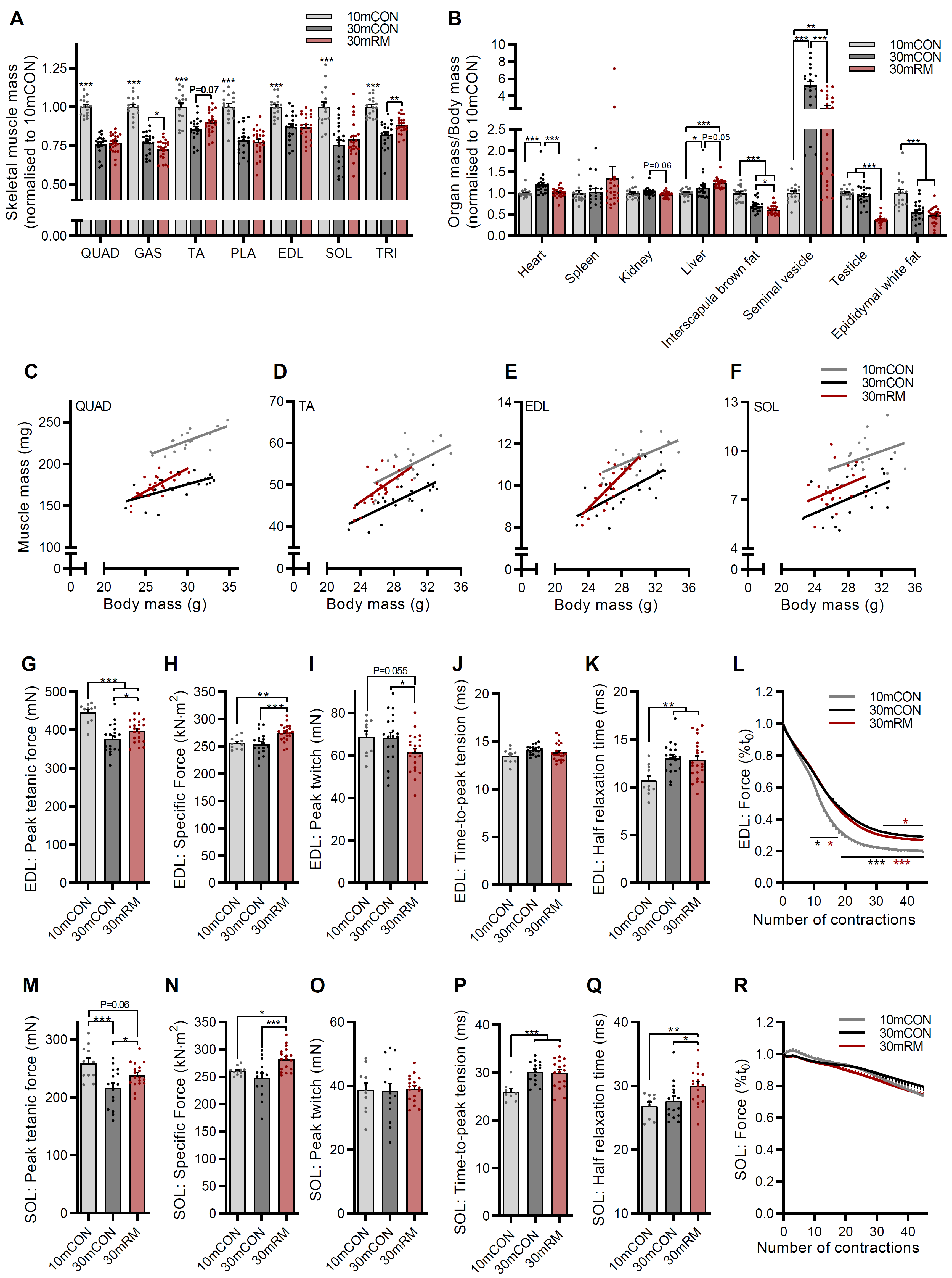


**Figure S2. Rapamycin has muscle- and organ-specific effects on mass and improves isolated muscle function**. (A) absolute limb muscle mass and (B) organ mass normalised to body mass and then 10mCON for visualisation purposes. Scatterplots and linear regressions showing the relationship between body mass and muscle mass is maintained, but both age and rapamycin effect muscle mass across the body mass spectrum in (C) *quadriceps* (QUAD), (D) *tibialis anterior* (TA), (E) *extensor digitorum longus* (EDL) and (F) *soleus* (SOL). Isolated muscle function parameters for (G-L) EDL and (M-R) SOL, including (G, M) absolute peak tetanic force, (H, N) specific force (peak tetanic force normalized to muscle cross sectional area; (I, O) peak twitch force; (J, P) time to peak twitch tension; (K, Q) twitch half relaxation time and; (L-R) the fatigue response to multiple stimulations normalized to starting force. Group numbers are (A-F) 17, 20 and 23 for 10mCON, 30mCON and 30mRM; (G-L) 8 (L), 10 (G-K), 19 and 22 for 10mCON, 30mCON and 30mRM and; (M-R) 9 (O-R), M-N (11), 14 (N) (M, O-R) and 18 (M-Q) 17 (R) for 10mCON, 30mCON and 30mRM. Data are presented as mean ± SEM. One-way ANOVA with Fisher’s LSD post hoc test (A-B, G-K, M-Q) and two-way repeated measures ANOVA with Tukey’s post hoc test (L, R) were used to compare between groups. *, ** and *** denote a significant difference between groups of P<0.05, P<0.01 and P<0.001, respectively. Where 0.05<P<0.1, P values are reported.

**Figure S3. Rapamycin-induced changes in IIA fiber size occurs before measureable loss of muscle mass.** Correlations between fiber type proportion and minimum fiber Feret in 30mCON and 30mRM mice for (A) IIA; (B) IIX and; (C) IIB fiber types. (D) Muscle mass in 21-month-old mice administered a control (21mCON) or rapamycin (21mRM) diet for 6 months compared with 10mCON mice. (E) Fiber type proportion and (F) minimum fiber feret in *tibialis anterior* muscle from 10mCON, 21mCON and 21mRM mice. (G) Representative images from the deep IIA-rich region of *tibialis anterior* muscle from each of the three 21mCON and 21mRM mice. (H) Fiber size distribution for IIA, IIX and IIB fibers from 10mCON, 21mCON and 21mRM mice. Sample numbers are (A-C) n=13 and 14 for 30mCON and 30mRM and (E-H) n=11, 3 and 3 for 10mCON, 21mCON and 21mRM. Data are presented as mean ± SEM. Pearson’s correlation (A-C) and one-way ANOVA with Fisher’s LSD post hoc test (D-F, H) were used to compare between data. *, ** and *** denote a significant difference between groups of P<0.05, P<0.01 and P<0.001, respectively.

**Figure S4. Multi-muscle fiber type-specific size**. Fiber type proportion and fiber type specific size distribution for (A-B) *extensor digitorum longus* (EDL); (C-D) *triceps brachii* (TRI) and; (E-F) soleus (SOL) in whole cross sections. For B, D and F between-group statistical comparisons were performed on the overall mean of the minimum fiber Feret. Group numbers are (A-B) 7, 9 and 8 for 10mCON, 30mCON and 30mRM, (C-D) 5, 9 and 9 for 10mCON, 30mCON and 30mRM and (E-F) 6, 6 and 7 for 10mCON, 30mCON and 30mRM. Data are presented as mean ± SEM. One-way ANOVA with Fisher’s LSD post hoc test were used to compare between data. *, ** and *** denote a significant difference between groups of P<0.05, P<0.01 and P<0.001, respectively. Where 0.05<P<0.1, P values are reported.





**Figure S5.** **Muscle and fiber type-specific prevalence of central nucleated fibers.** (A) Representative images of fiber type-specific centro-nucleated fibers in IIB-rich (above) and IIA-rich (below) regions of *tibialis anterior muscle* taken from n=9, 10 and 10 mice from 10mCON, 30mCON and 30mRM groups Centro-nucleated fibers are denoted by white arrows. (B) Quantification of fiber-type specific centro-nucleated fibers in (B) *extensor digitorum longus* (EDL) and (C) *soleus* (SOL) muscles. Sample numbers are (A) n=9, 10 and 10, (B) n=7, 9 and 8 and (C) 6, 6 and 7 for 10mCON, 30mCON and 30mRM, respectively. Data are presented as mean ± SEM. One-way ANOVA with Fisher’s LSD post hoc test were used to compare between data. *, ** and *** denote a significant difference between groups of P<0.05, P<0.01 and P<0.001, respectively. Where 0.05<P<0.1, P values are reported.

**Figure S6. In vitro responses to rapamycin*.*** (A) Quantification of P62 protein abundance in 10mCON, 30mCON and 30mRM in both the *tibialis anterior* (TA) and *gastrocnemius* (GAS) muscle. (B) Quantification of C2C12 myotube diameter after 24 h incubation in differentiation media (CON) or media containing 10 nM rapamycin, 20 ng·ml^-1^ TNFα and 100 ng·ml^-1^ IFNγ (cytokines) or cytokines and rapamycin (cytokines+RM). Data are normalised to the CON group only. For each biological replicate, 40+ myotubes were measured across 4 images and averaged. In 5-day-old myotubes, 24 h incubation with rapamycin impairs (C) myonuclei accretion and (D) myotube hypertrophy. (E) Representative images of C2C12 myoblasts incubated in differentiation media with or without rapamycin for 5 days shows dose-dependent impairment of myotube formation by rapamycin. Group numbers are n=7 mice (10mCON), 8 (30mCON), 7 for TA and 8 for GAS (30RM), n=6 (B) and n=3 (C-E). Data are presented as mean ± SEM. Two-way ANOVA with Sidak’s post hoc test (A) and one-way ANOVA with Tukey’s post hoc test (B-D) were used to compare between groups. *, ** and *** denote a significant difference between groups of P<0.05, P<0.01 and P<0.001, respectively. Where 0.05<P<0.1, P values are reported.


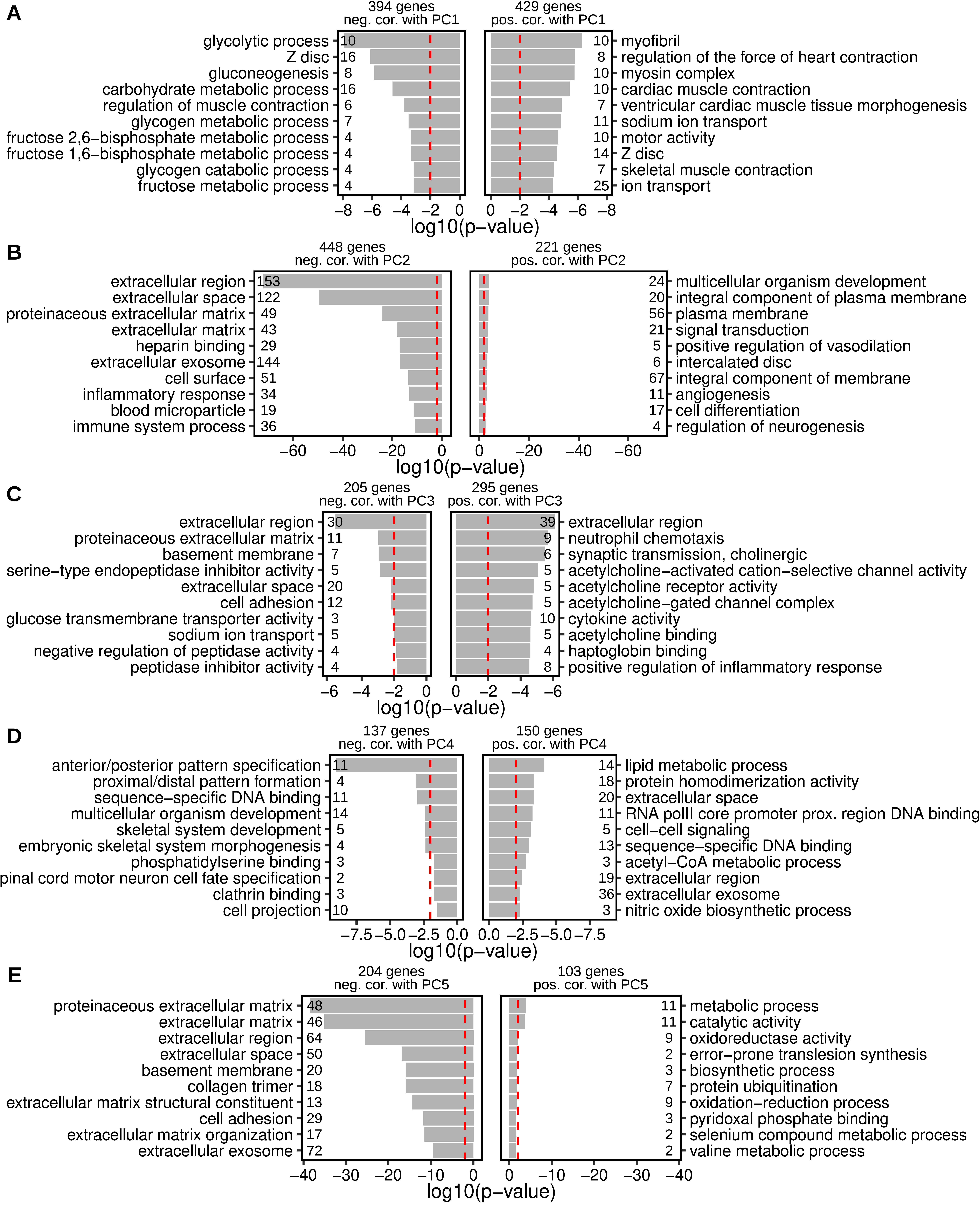


**Figure S7. GO analysis of PCs 1-5.** Top 10 gene ontology (GO) terms enriched for genes positively and negatively aligned with principal components (PCs) (A) 1; (B) 2; (C) 3; (D) 4 and; (E) 5 for 10mCON, 30mCON and 30mRM data sets from Figure 5C. The number of genes aligned with PCs and used for GO enrichment analysis (DAVID) is indicated at the top of each panel. We considered P<0.01 (red dashed line) as the significance threshold for enrichment. The number of genes contributing to each GO term is indicated next to the GO term labels. Sample numbers are n=6 mice for each group and each muscle (i.e. 72 samples). A modified Fisher’s exact test was used to determine significance.


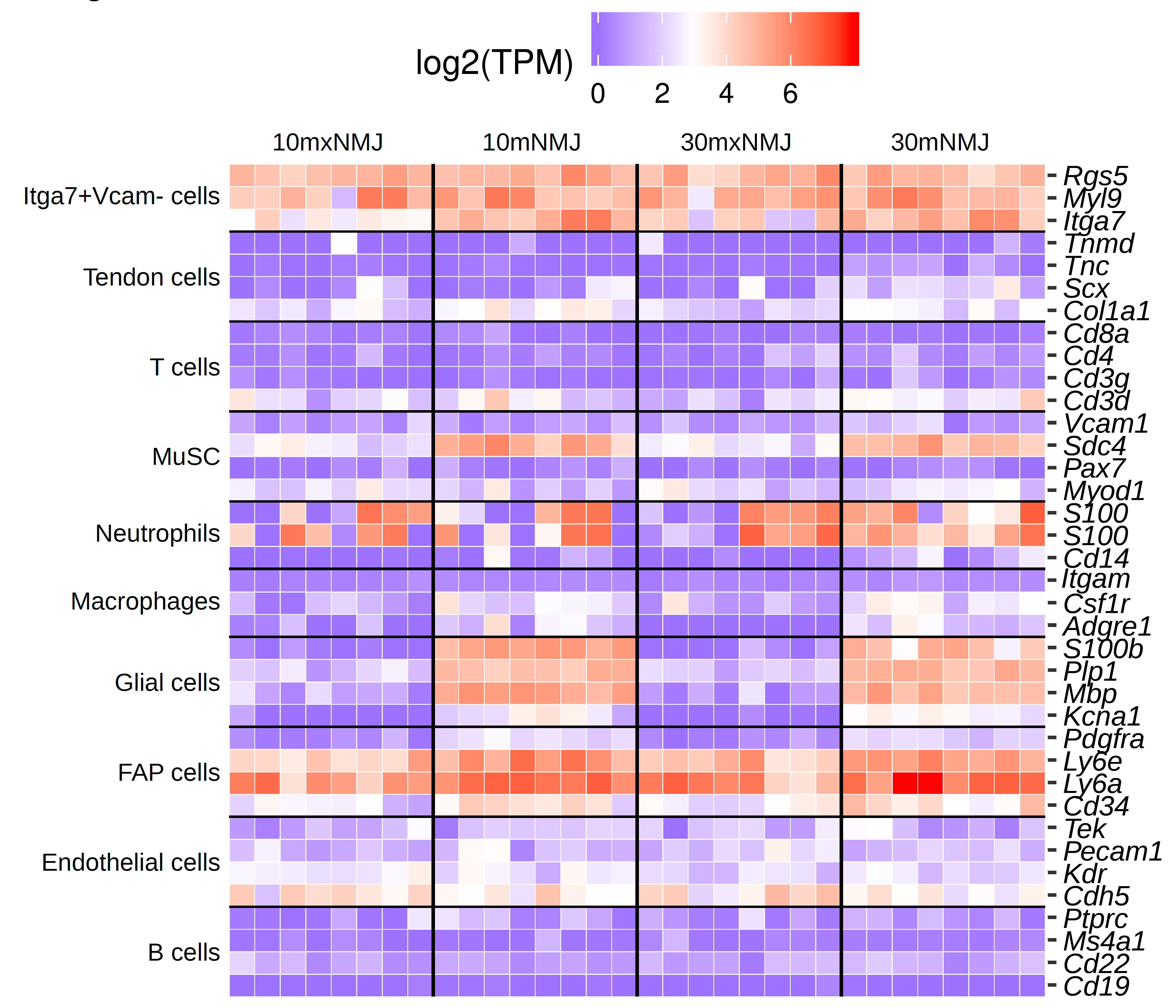


**Figure S8. Markers of glial, but not other cell types, are increased at the NMJ.** The expression pattern of known cell type markers in skeletal muscle in synaptic (NMJ) and extra synaptic (xNMJ) regions collected via laser capture microdissection in *tibialis anterior* muscle from 10mCON and 30mCON mice.


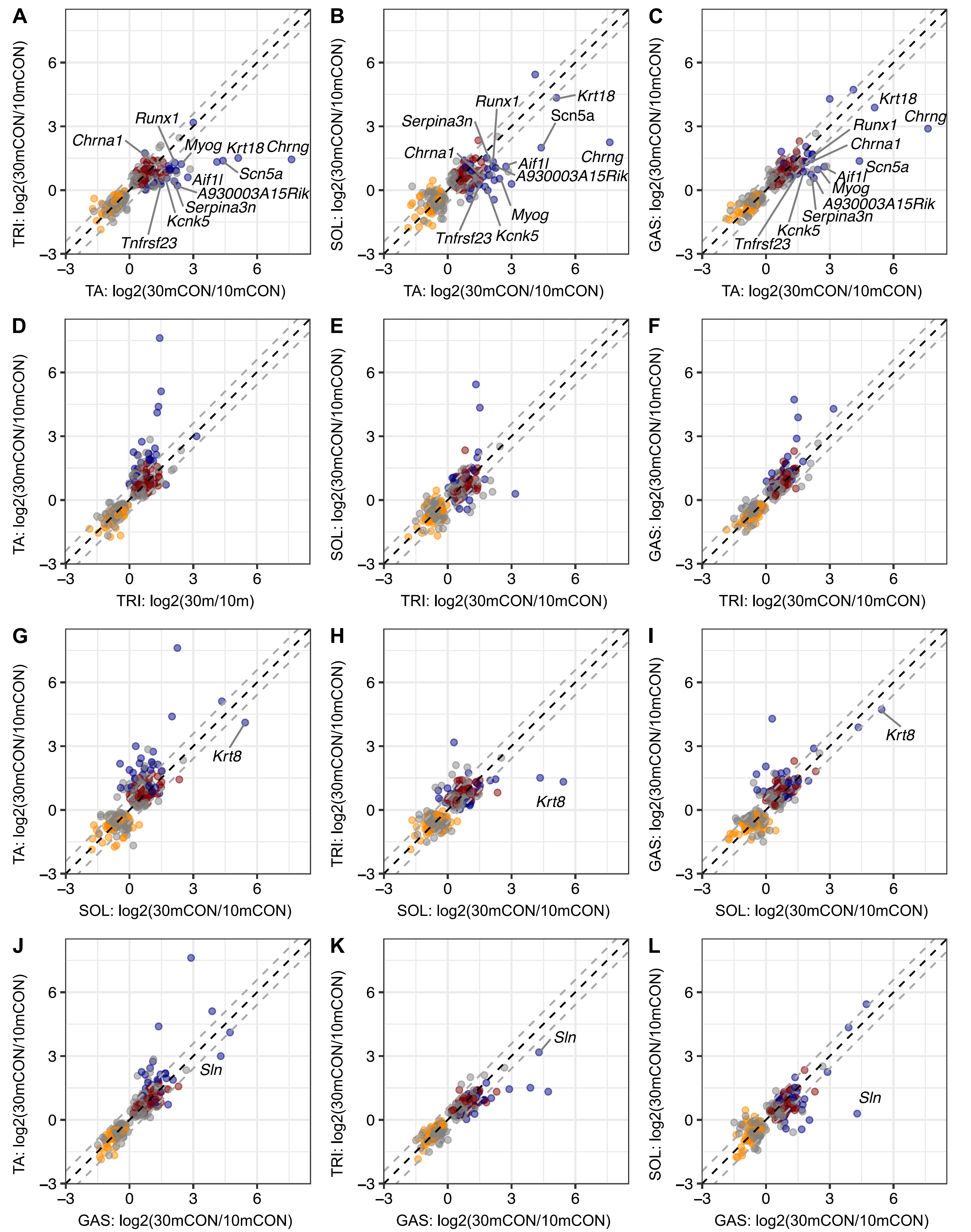


**Figure S9. Tibialis anterior has an exaggerated ‘denervation’ gene expression response to aging**. Scatterplots of fold-change gene expression from natural aging PC3 between 30mCON and 10mCON for (A-C) *tibialis anterior* (TA), (D-F) *triceps brachii* (TRI), (G-I) *soleus* (SOL) and (J-L) *gastrocnemius* (GAS) and the other three muscles. Genes from clusters 1, 2 and 3 are colored orange, red and blue, respectively, while gray dots are genes aligned with aging PC3, but not belonging to clusters 1-3. Labelled genes show a > log_2_(1.5) fold change between one muscle and all three other muscles. The dashed black line represents a 1:1 relationship in fold-change gene expression between muscles, while the gray dashed lines represent a difference of log_2_(1.5) fold between muscles.


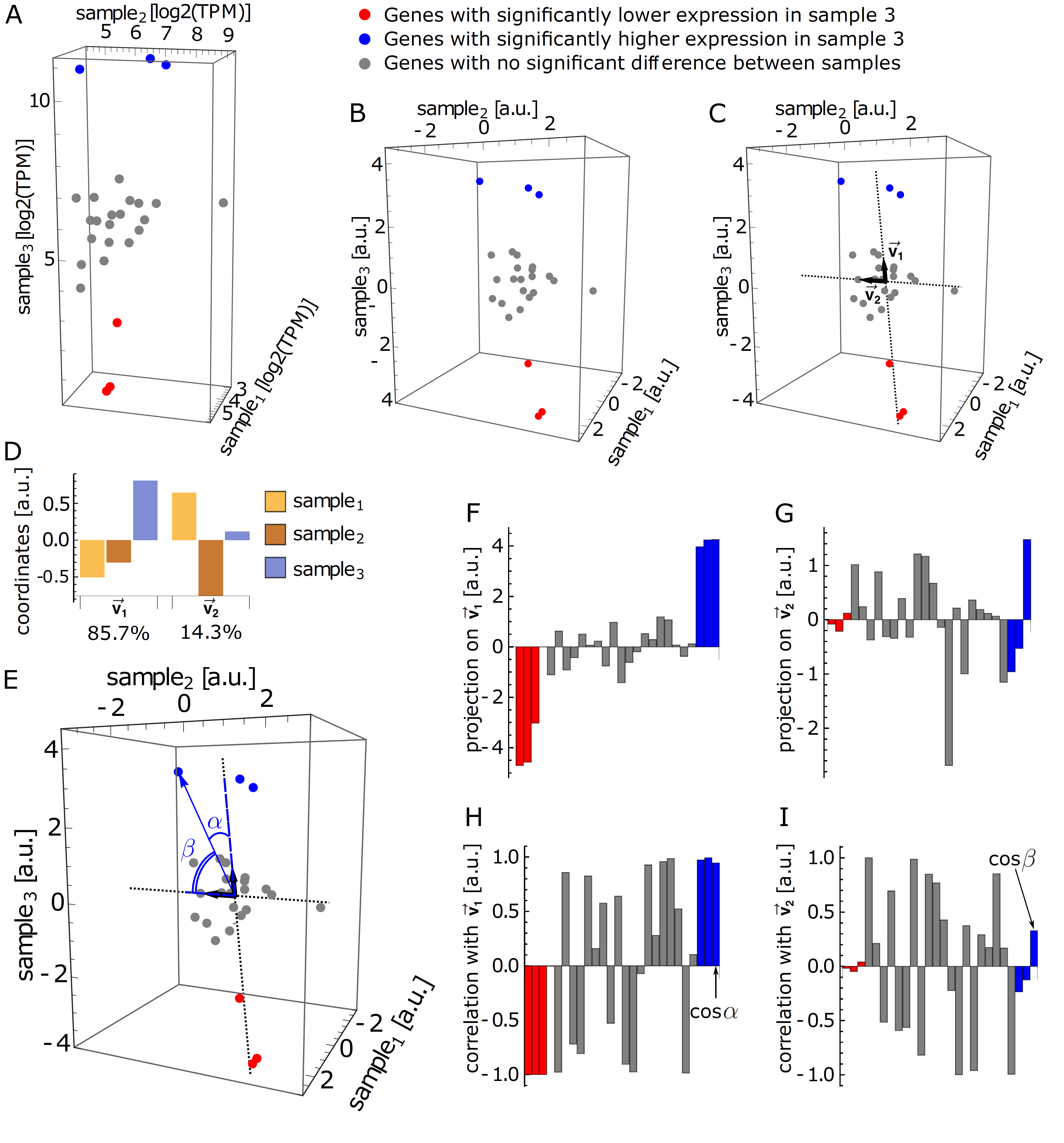


**Figure S10. Application of singular value decomposition in the analysis of gene expression**. (A) Visualisation of a toy data set in sample space with 3 samples and 26 genes. Each dot corresponds to a gene, the coordinates being the expression level of the gene in each of the three samples in log space. Grey dots correspond to genes with similar expression across all samples, while red and blue dots correspond, to genes that have significantly lower and higher expression in sample 3 compared to the other two samples, respectively. (B) Similar visualization of the centered gene expression data. (C-D) The first two principal components, $\vec{v}_{1}$ and $\vec{v}_{2}$ (black arrows), defining the directions (dotted lines) of highest variance between samples, and their corresponding coordinates in sample space. (E) Visualization of projections (blue dashed lines) of a representative gene vector (blue arrow) on the first two PCs with the corresponding angles $\alpha$ and $\beta$ between gene vector and PCs. Projections of gene vectors on (F) PC $\vec{v}_{1}$ and (G) PC $\vec{v}_{2}$. Pearson correlation coefficients of gene vectors with (H) PC $\vec{v}_{1}$ and (I) PC $\vec{v}_{2}$. For (F-I) each bar corresponds to a gene. Bars are colored as corresponding genes in A-C and E, with the order of genes preserved across all panels.
