# Supplementary material for: The neuromuscular junction is a focal point of mTORC1 signaling in sarcopenia
Source: Nat Commun. 2020 Sep 9;11:4510. doi: 10.1038/s41467-020-18140-1 (PMC7481251; doi:10.1038/s41467-020-18140-1)
Supplement: Supplementary file 2 — Reporting Summary [file 41467_2020_18140_MOESM2_ESM.pdf]

nature research

Corresponding author(s): Markus A. Rüegg

Last updated by author(s): Jun 3, 2020

Reporting Summary

Nature Research wishes to improve the reproducibility of the work that we publish. This form provides structure for consistency and transparency in reporting. For further information on Nature Research policies, see our Editorial Policies and the Editorial Policy Checklist.

Statistics

For all statistical analyses, confirm that the following items are present in the figure legend, table legend, main text, or Methods section.

n/a	Confirmed	
☐	☒	The exact sample size (*n*) for each experimental group/condition, given as a discrete number and unit of measurement
☐	☒	A statement on whether measurements were taken from distinct samples or whether the same sample was measured repeatedly
☐	☒	The statistical test(s) used AND whether they are one- or two-sided<br>*Only common tests should be described solely by name; describe more complex techniques in the Methods section.*
☒	☐	A description of all covariates tested
☐	☒	A description of any assumptions or corrections, such as tests of normality and adjustment for multiple comparisons
☐	☒	A full description of the statistical parameters including central tendency (e.g. means) or other basic estimates (e.g. regression coefficient) AND variation (e.g. standard deviation) or associated estimates of uncertainty (e.g. confidence intervals)
☐	☒	For null hypothesis testing, the test statistic (e.g. *F*, *t*, *r*) with confidence intervals, effect sizes, degrees of freedom and *P* value noted<br>*Give P values as exact values whenever suitable.*
☒	☐	For Bayesian analysis, information on the choice of priors and Markov chain Monte Carlo settings
☐	☒	For hierarchical and complex designs, identification of the appropriate level for tests and full reporting of outcomes
☐	☒	Estimates of effect sizes (e.g. Cohen's *d*, Pearson's *r*), indicating how they were calculated

Our web collection on statistics for biologists contains articles on many of the points above.

Software and code

Policy information about availability of computer code

Data collection	RNAseq data were processed using Cutadapt v1.9.1 and Kallisto v0.43.1. Sequences of protein coding transcripts were selected based on genome assembly GRCm38 (release 92) and transcript annotations from Ensembl database. RNAseq data were analysed using the Bioconductor packages EdgeR v3.26.1 and RDAVIDWebService v1.22. Gene set enrichment analysis was performed using GSEA v2.2.4. SarcoAtlas (https://sarcoatlas.scicore.unibas.ch/) was developed using the R package Shiny v0.14.2.
Data analysis	Data were analysed using GraphPad Prism 8.0.2.

For manuscripts utilizing custom algorithms or software that are central to the research but not yet described in published literature, software must be made available to editors and reviewers. We strongly encourage code deposition in a community repository (e.g. GitHub). See the Nature Research guidelines for submitting code & software for further information.

Data

Policy information about availability of data

All manuscripts must include a data availability statement. This statement should provide the following information, where applicable:

- Accession codes, unique identifiers, or web links for publicly available datasets
- A list of figures that have associated raw data
- A description of any restrictions on data availability

Raw and processed RNAseq data are available at Gene Expression Omnibus (GEO)95 (https://www.ncbi.nlm.nih.gov/geo/) under accession number GSE139214 (SubSeries GSE139204, GSE139209 and GSE139213). These data are also accessible using the web-based application, SarcoAtlas (https://sarcoatlas.scicore.unibas.ch/). Source data are provided with this paper.

Field-specific reporting

Please select the one below that is the best fit for your research. If you are not sure, read the appropriate sections before making your selection.

- [x] Life sciences
- [ ] Behavioural & social sciences
- [ ] Ecological, evolutionary & environmental sciences

For a reference copy of the document with all sections, see nature.com/documents/nr-reporting-summary-flat.pdf

Life sciences study design

All studies must disclose on these points even when the disclosure is negative.

Sample size	For TSCmKO mice, sample sizes were based on previous experience in the lab (Castets, Cell Metabolism, 2013). For long-term rapamycin studies, sample sizes took into consideration expected survival rates (~50%) along with expected variability in the extent of sarcopenia between mice.
Data exclusions	A single outlier in the 30mCON SOL group was identified in mRNA sequencing analysis and removed from further analysis based on a clear technical error.
Replication	Phenotypical responses (body mass, body composition, grip strength and voluntary running distance) to aging and rapamycin were examined in two independent experiments starting at 15 and 20 months of age. Results were reproducible between experiments (see figure 1). The effect of rapamycin and cytokines were examined across two independent experiments, with reproducible results. All attempts at replication were successful.
Randomization	Treatment groups were balanced based on pre-treatment measures of body mass, body composition, food intake and functional parameters.
Blinding	In NMJ transmission experiments using TSCmKO mice and for in vitro measurements of muscle force in long-term rapamycin studies researchers were blinded for genotype. Due to technical/researcher limitations and obvious phenotypes, blinding was not performed for all other experiments.

Reporting for specific materials, systems and methods

We require information from authors about some types of materials, experimental systems and methods used in many studies. Here, indicate whether each material, system or method listed is relevant to your study. If you are not sure if a list item applies to your research, read the appropriate section before selecting a response.

Materials & experimental systems

n/a	Involved in the study	
☐	☒	Antibodies
☐	☒	Eukaryotic cell lines
☒	☐	Palaeontology and archaeology
☐	☒	Animals and other organisms
☒	☐	Human research participants
☒	☐	Clinical data
☒	☐	Dual use research of concern

Methods

n/a	Involved in the study	
☒	☐	ChIP-seq
☒	☐	Flow cytometry
☒	☐	MRI-based neuroimaging

Antibodies

Antibodies used	α-actinin mouse IgG1 EA-53 monoclonal (WB: 1:5000), Sigma A7732; Akt rabbit polyclonal (WB: 1:1000), Cell Signaling 9272, p-AktT308 rabbit polyclonal (WB: 1:1000), Cell Signaling 9275; p62 guinea pig polyclonal (WB: 1:1000), Progen GP62-C; S6 rabbit IgG monoclonal (WB: 1:1000), Cell Signaling 2217; p-S6S240/244 rabbit IgG monoclonal (WB: 1:1000), Cell Signaling 5364; 4E-BP1 rabbit polyclonal (WB: 1:1000), Cell Signaling 9452; p-4E-BP1T37/46 rabbit polyclonal (WB: 1:1000), Cell Signaling 9459; GAPDH rabbit monoclonal (WB 1:1000), Cell Signaling 2118; ATF4 rabbit polyclonal (WB: 1:1000), Santa Cruz sc-200; Desmin (IHC: 1:300), Abcam Ab15200; p-S6S235/236 (IHC: 1:100), Cell Signaling 2211; Laminin 2α (IHC: 1:100), Abcam 11576; Myosin 7 (IHC: 1:50), DSHB BA-D5; Myosin 2 (IHC: 1:200), DSHB SC-71; Myosin 4 (IHC: 1:50), DSHB BF-F3; Laminin (IHC: 1:150), Abcam 11575; Alexa647 Donkey anti-Rabbit IgG polyclonal, Jackson #711-605-152; α-bungarotoxin Alexa555-conjugate, Invitrogen #B35451; α-bungarotoxin Alexa488 conjugate, Invitrogen #B13422; Alexa488 Goat anti-rat IgG, Jackson #112-545-003; Alexa488 Goat anti-mouse IgG2b, Invitrogen #A-21141; Alexa488 Goat anti-mouse IgM, Invitrogen #A-21042; DyLight 405 Goat anti-mouse IgG2b, Jackson Immuno #115-475-207; Alexa568 Goat anti-mouse IgG1, Invitrogen #A-21124; HRP goat anti-mouse (1:10000), Jackson Immuno 115-035-003; HRP goat anti-rabbit (1:10000), Jackson Immuno 111-035-003; HRP donkey anti-guinea pig (1:10000), Jackson Immuno 706-035-148.
Validation	α-actinin mouse IgG1 EA-53 monoclonal (WB: 1:5000), Sigma A7732; https://www.sigmaaldrich.com/catalog/product/sigma/a7732?lang=en&region=CH<br>Akt rabbit polyclonal (WB: 1:1000), Cell Signaling 9272; https://www.cellsignal.com/products/primary-antibodies/akt-antibody/9272<br>p-AktT308 rabbit polyclonal (WB: 1:1000), Cell Signaling 9275; https://www.cellsignal.com/products/primary-antibodies/phospho-

akt-thr308-antibody/9275
p62 guinea pig polyclonal (WB: 1:1000), Progen GP62-C; https://www.progen.com/anti-p62-sqstm1-c-terminus-guinea-pig-polyclonal-serum.html
S6 rabbit IgG monoclonal (WB: 1:1000), Cell Signaling 2217; https://www.cellsignal.com/products/primary-antibodies/s6-ribosomal-protein-5g10-rabbit-mab/2217
p-S6S240/244 rabbit IgG monoclonal (WB: 1:1000), Cell Signaling 5364; https://www.cellsignal.com/products/primary-antibodies/phospho-s6-ribosomal-protein-ser240-244-d68f8-xp-rabbit-mab/5364
4E-BP1 rabbit polyclonal (WB: 1:1000), Cell Signaling 9452; https://www.cellsignal.com/products/primary-antibodies/4e-bp1-antibody/9452
p-4E-BP1T37/46 rabbit polyclonal (WB: 1:1000), Cell Signaling 9459; https://www.cellsignal.com/products/primary-antibodies/phospho-4e-bp1-thr37-46-antibody/9459
GAPDH rabbit monoclonal (WB 1:1000), Cell Signaling 2118; https://www.cellsignal.com/products/primary-antibodies/gapdh-14c10-rabbit-mab/2118
ATF4 rabbit polyclonal (WB: 1:1000), Santa Cruz sc-200; https://www.citeab.com/antibodies/783974-sc-200-creb-2-antibody-c-20
Desmin (IHC: 1:300), Abcam Ab15200; https://www.abcam.com/desmin-antibody-cytoskeleton-marker-ab15200.html
p-S6S235/236 (IHC: 1:100), Cell Signaling 2211; https://www.cellsignal.com/products/primary-antibodies/phospho-s6-ribosomal-protein-ser235-236-antibody/2211
Laminin 2α (IHC: 1:100), abcam 11576; https://www.abcam.com/laminin-2-alpha-antibody-4h8-2-ab11576.html
Myosin 7 (IHC: 1:50), DSHB BA-D5; https://dshb.biology.uiowa.edu/BA-D5
Myosin 2 (IHC: 1:200), DSHB SC-71; https://dshb.biology.uiowa.edu/SC-71
Myosin 4 (IHC: 1:50), DSHB BF-F3; https://dshb.biology.uiowa.edu/BF-F3
Laminin (IHC: 1:150), abcam 11575; https://www.abcam.com/laminin-antibody-ab11575.html
Alexa647 Donkey anti-Rabbit IgG polyclonal, Jackson #711-605-152; https://www.jacksonimmuno.com/catalog/products/711-605-152
α-bungarotoxin Alexa555-conjugate, Invitrogen #B35451; https://www.thermofisher.com/order/catalog/product/B35451#/B35451
α-bungarotoxin Alexa488 conjugate, Invitrogen #B13422; https://www.thermofisher.com/order/catalog/product/B13422#/B13422
Alexa488 Goat anti-rat IgG, Jackson #112-545-003; https://www.jacksonimmuno.com/catalog/products/112-545-003
Alexa488 Goat anti-mouse IgG2b, Invitrogen #A-21141; https://www.thermofisher.com/antibody/product/Goat-anti-Mouse-IgG2b-Cross-Adsorbed-Secondary-Antibody-Polyclonal/A-21141
Alexa488 Goat anti-mouse IgM, Invitrogen #A-21042; https://www.thermofisher.com/antibody/product/Goat-anti-Mouse-IgM-Heavy-chain-Cross-Adsorbed-Secondary-Antibody-Polyclonal/A-21042
DyLight 405 Goat anti-mouse IgG2b, Jackson Immuno #115-475-207; https://www.jacksonimmuno.com/catalog/products/115-475-207
Alexa568 Goat anti-mouse IgG1, Invitrogen #A-21124; https://www.thermofisher.com/antibody/product/Goat-anti-Mouse-IgG1-Cross-Adsorbed-Secondary-Antibody-Polyclonal/A-21124
HRP goat anti-mouse (1:10000), Jackson Immuno 115-035-003; https://www.jacksonimmuno.com/catalog/products/115-035-003
HRP goat anti-rabbit (1:10000), Jackson Immuno 111-035-003; https://www.jacksonimmuno.com/catalog/products/111-035-003
HRP donkey anti-guinea pig (1:10000), Jackson Immuno 706-035-148; https://www.jacksonimmuno.com/catalog/products/706-035-148

Eukaryotic cell lines

Policy information about cell lines

Cell line source(s)	C2C12 myoblasts/myotubes from ATCC
Authentication	Cells were authenticated by confirming their fusion into myotubes upon differentiation stimulus.
Mycoplasma contamination	Mycoplasma screening was not performed.
Commonly misidentified lines (See ICLAC register)	None

Animals and other organisms

Policy information about studies involving animals; ARRIVE guidelines recommended for reporting animal research

Laboratory animals	C57BL/6JRj mice were purchase from the aging colony at Janvier Labs. TSCmKO mice and WT controls were bred on a C57BL/6JRj background. All mice were males, except for the denervation study, which contained both male and female mice. Wild-type mice used for long term rapamycin studies were examined between 8 and 31 months of age. TSCmKO mice were 3-12 months old. Denervated mice were 5-14 months old. Mice were kept on a 12 hr light-dark cycle (6 am to 6 pm) at 22°C (range 20-24°C) and 55% (range 45-65%) relative humidity
Wild animals	This study did not involve wild animals
Field-collected samples	This study did not involve field-collected samples
Ethics oversight	All experiments were approved by the regional animal ethics Committee of Basel-Stadt, Switzerland.

Note that full information on the approval of the study protocol must also be provided in the manuscript.
